# Decoding the CAF–TAM axis: multi-omics dissection and therapeutic targeting of stromal–immune crosstalk in the tumor microenvironment

**DOI:** 10.1038/s41419-026-08685-2

**Published:** 2026-04-18

**Authors:** Yage Fu, Mei Li, Shiwang Wu, Jingsi Wang, Shan Wang

**Affiliations:** Department of Oral Pathology, School of Stomatology, Hainan Medical University, Haikou, 571199 P. R. China

**Keywords:** Cell signalling, Translational research

## Abstract

Cancer-associated fibroblasts (CAFs) and tumor-associated macrophages (TAMs) are pivotal stromal and immune components of the tumor microenvironment (TME) that orchestrate immune evasion, metabolic reprogramming, and therapeutic resistance. Regions enriched in CAFs are frequently accompanied by dense TAM infiltration, underscoring their close structural and functional interdependence. Recent advances in single-cell, multi-omics, and spatial profiling technologies have revolutionized our understanding of the spatiotemporal heterogeneity, lineage evolution, and bidirectional signaling between CAFs and TAMs. In this review, we summarize the latest progress from transcriptomic, epigenomic, metabolomic, and spatial multi-omics studies, highlighting the diverse CAF/TAM subpopulations, their intercellular communication networks, and their collective roles in extracellular matrix remodeling, immune suppression, metabolic adaptation, and angiogenesis. Finally, we discuss emerging therapeutic strategies targeting CAFs, TAMs, and their interactive pathways, offering new conceptual and translational insights into reprogramming the immunosuppressive TME and enhancing antitumor efficacy.

## FACTS

- The CAF–TAM axis is a central driver of immune evasion and therapeutic resistance in the tumor microenvironment: Despite extensive single-cell and spatial multi-omics data, the signaling networks, metabolic interplay, and spatial organization between CAFs and TAMs remain incompletely decoded.
- Heterogeneity and plasticity of CAF/TAM subsets require deeper elucidation: Emerging populations such as LRRC15⁺ myCAFs, and TREM2⁺ LAMs display tumor type–specific and dynamic functions, yet their origins, differentiation trajectories, and intercellular mechanisms remain controversial.
- Metabolic coupling represents a crucial but underexplored dimension of CAF–TAM crosstalk: Lactate shuttling, lipid transfer, and amino-acid reprogramming may constitute novel therapeutic targets, but their spatiotemporal dynamics and adaptive responses under treatment remain undefined.
- Spatial topology dictates the formation of immunosuppressive niches: Multi-omics studies suggest that co-localization of CAFs and TAMs at invasive fronts contributes to immune barriers, but how these structures evolve across tumor types and treatment contexts is still unclear.
- Future directions must focus on dynamics and integration: Leveraging multimodal omics, real-time imaging, and AI-based modeling to reconstruct dynamic CAF–TAM networks and optimize therapeutic combinations and timing represents a critical challenge for the field.

## Introduction

The tumor microenvironment (TME) is not merely a bystander in cancer development but a dynamic and evolving ecosystem that orchestrates immune suppression, metabolic adaptation, and therapeutic resistance. Composed of neoplastic, immune, stromal, and vascular elements, the TME evolves in a spatiotemporal manner through a complex web of interactions governed by mechanical, metabolic, and epigenetic cues [1–5]. Among these, the reciprocal crosstalk between cancer-associated fibroblasts (CAFs) and tumor-associated macrophages (TAMs) has emerged as a pivotal axis driving malignant progression and therapeutic failure [6–8].

CAFs, as the most plastic stromal cells, reshape the tumor niche through extracellular matrix (ECM) remodeling, immune exclusion, and metabolic rewiring. By secreting collagen (e.g., COL1A1) and MMPs, CAFs modulate ECM stiffness to create a pro-metastatic biomechanical gradient [4]. Importantly, CAFs operate within a three-dimensional, multicellular interaction network that includes tumor epithelial cells, endothelial cells, pericytes, and diverse immune subsets; this 3D architecture enables CAFs to coordinate spatial patterning of growth, angiogenesis, and immunosuppression [8]. Functionally validated examples illustrate the multi-directional nature of these interactions. In gastric cancer (GC), CAF-secreted FGF2 promotes tumor-cell proliferation through FGFR1-dependent ribosome biogenesis, providing a clear paracrine mechanism of CAF-driven growth [9]. Additional studies in GC and other tumor types demonstrate that CAF infiltration can activate TGF-β–driven epithelial-mesenchymal transition (EMT) and metastasis [10], while in triple-negative breast cancer (TNBC), tumor-cell-derived EMP1 enhances IL-6 production and thereby recruits and activates CAFs, establishing a reciprocal tumor cell–CAF loop [11]. Collectively, these studies provide experimental evidence that CAF–tumor cell crosstalk can causally drive proliferation, EMT, and stromal recruitment. Simultaneously, cytokines such as CXCL12 and TGF-β enforce immune privilege by recruiting regulatory T cells (Tregs) and suppressing cytotoxic T lymphocytes (CTLs) [8]. TAMs, in turn, are the most abundant immune component in CAF–rich areas and frequently adopt immunosuppressive phenotypes. For instance, SPP1⁺ TAMs promote cancer stemness via IGF1 and collagen signaling and correlate with resistance to PD-L1 blockade [12]. Recent spatial transcriptomic analyses reveal that FAP⁺ CAFs and SPP1⁺ TAMs co-localize to form immunosuppressive microniches at the tumor invasive front [13, 14]—raising the question of how these cellular compartments coordinate to drive immune evasion and therapeutic failure.

Advances in single-cell and spatial omics technologies have opened unprecedented opportunities to address this question. Platforms such as single-cell RNA sequencing (scRNA-seq), single-cell assay for transposase-accessible chromatin using sequencing (scATAC-seq), spatial transcriptomics (ST), and integrative multimodal methods such as cellular indexing of transcriptomes and epitopes by sequencing (CITE-seq) and transposable enzyme–accessible chromatin sequencing (TEA-seq) now enable simultaneous profiling of transcriptomes, chromatin landscapes, surface proteomes, and epigenetic states within spatially defined cellular contexts [15–19]. These innovations have led to the discovery of distinct CAF and TAM subpopulations—including LRRC15⁺ myofibroblastic CAFs (myCAFs), T cell–inhibitory CAF subsets (TinCAFs) [20], TREM2⁺ lipid-associated macrophages (LAMs), and FOLR2⁺ M2-like TAMs—that carry context-specific immunometabolic phenotypes and are unevenly distributed across tumor zones [21, 22].

Yet despite the explosion of descriptive data, several key conceptual gaps remain. First, the bidirectional signaling circuits between CAFs and TAMs—such as CXCL12–CXCR4, IL-6–STAT3, and ECM–integrin–FAK pathways—are incompletely mapped across tumor types and stages [6]. Second, the temporal dynamics of their interactions under treatment pressure—chemotherapy, immune-checkpoint blockade, or radiotherapy—remain largely undefined. Third, the metabolic co-dependence between CAFs and TAMs, including lactate shuttling, arginine/nitric oxide modulation, and lipid crosstalk, is poorly understood but may represent a therapeutically actionable vulnerability. In this review, we integrate current transcriptomic, spatial, metabolic, and epigenetic evidence to delineate the heterogeneity and functional crosstalk of CAFs and TAMs, and to define how this axis orchestrates immune evasion, ECM remodeling, and resistance to therapy. We further outline emerging therapeutic strategies targeting CAFs, TAMs, and their shared signaling circuitry—including CSF1R and FAP inhibition, stromal reprogramming with ATRA and vitamin D, metabolic rewiring, and CAR-macrophage (CAR-M) therapies [23–25]—thereby providing a framework for rational combinations that reprogram the immunosuppressive TME. Understanding the CAF–TAM axis not only holds the key to unlocking stromal immune evasion but also serves as a lens to re-envision therapeutic timing and combinatorial design. Future directions will require AI-guided modeling of multicellular networks [26], multi-omics integration pipelines [18, 27], and real-time imaging to capture the plasticity of these interactions in situ [28, 29]. Only by shifting from static stromal depletion to dynamic immunomodulation can we realize intelligent remodeling of the TME.

## CAF heterogeneity: unveiling diverse subtypes and functional spectrum heterogeneity of CAF subtypes revealed by single-cell and spatial transcriptomics

Because CAFs arise from multiple precursor cell types, tumors typically harbor heterogeneous fibroblast populations whose phenotypes and functions vary across cancer entities [30, 31]. Despite differences in nomenclature across studies, a broad consensus has emerged from scRNA-seq and spatial transcriptomic surveys: three major CAF programs—myofibroblastic CAFs (myCAFs), inflammatory CAFs (iCAFs), and antigen-presenting CAFs (apCAFs)—recur across most solid tumors, alongside several less common lineage states [31]. Öhlund et al. first defined the canonical myCAF and iCAF dichotomy in pancreatic ductal adenocarcinoma (PDAC), demonstrating distinct spatial localization, transcriptional programs, and functional roles [32]. Subsequent studies have confirmed similar CAF diversity in bladder cancer (BlCa), cervical cancer (CC), esophageal cancer (EC), clear cell renal-cell carcinoma (ccRCC), lung adenocarcinoma (LUAD), prostate cancer (PCa), melanoma (MEL), colorectal cancer (CRC), and breast cancer (BC) [31, 33–38]. Beyond descriptive scRNA-seq clustering, recent work in ovarian cancer (OC) has demonstrated that CAF states correspond to functionally validated stromal programs rather than transcriptional artifacts. Using integrated scRNA-seq, spatial proteomics, and TCF21 perturbation, investigators identified three stable CAF states—matrix-remodeling, cytokine-secreting, and antigen-presenting—and showed that TCF21 directly regulates ECM remodeling and immune-modulating gene programs, thereby altering CD8⁺ T cell infiltration and clinical outcome. Functional assays confirmed that TCF21 loss reduces collagen deposition and disrupts fibroblast–immune interactions, providing causal evidence that specific CAF signatures correspond to discrete biological activities [39].

**Fig. 1:**
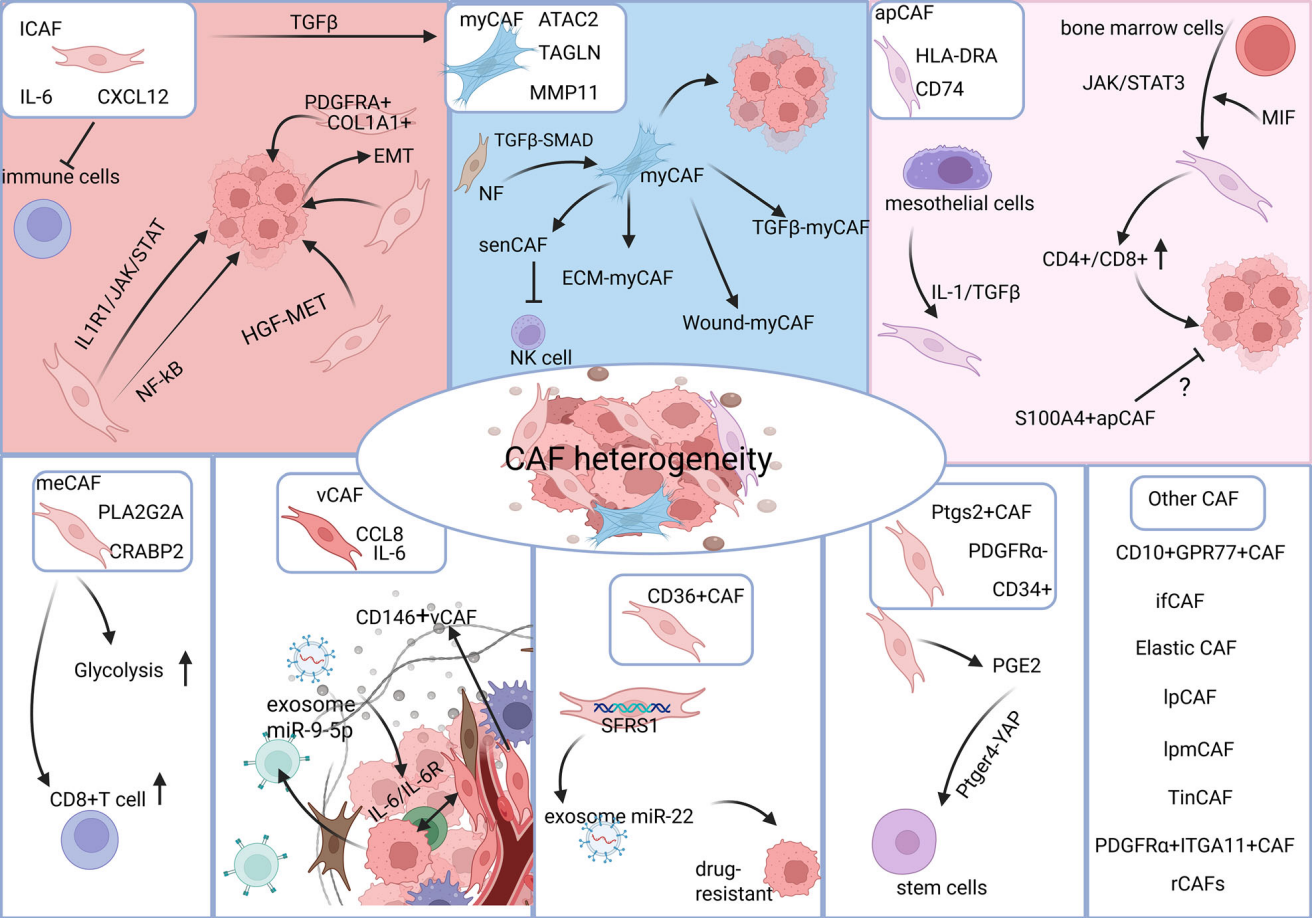
Functional heterogeneity and regulatory programs of cancer-associated fibroblast (CAF) subtypes in the tumor microenvironment. CAFs exhibit marked heterogeneity and can be classified into multiple functionally distinct subtypes. Inflammatory CAFs (iCAFs) are characterized by high secretion of IL-6 and CXCL12, with their differentiation driven by coordinated activation of the IL1R1/JAK/STAT and NF-κB pathways. iCAFs suppress CD8⁺ T cell cytotoxicity and establish an immunosuppressive niche, while also promoting ductal tumor growth through the HGF–MET axis. A COL1A1⁺PDGFRA⁺ iCAF subset facilitates tumor progression via ECM remodeling, and iCAFs overall enhance cancer progression by stimulating proliferation, EMT, and immune evasion. MyCAFs show high expression of ACTA2 and TAGLN and are enriched in matrix-remodeling genes such as MMP11. TGF-β signaling induces the transition from normal fibroblasts (NFs) to myCAFs through the SMAD pathway. A senescent subpopulation of myCAFs (senCAFs) inhibits NK cell cytotoxicity via ECM secretion. myCAFs can be further divided into ECM-myCAFs, TGFβ-myCAFs, and Wound-myCAFs based on functional signatures. Antigen-presenting CAFs (apCAFs) are defined by elevated expression of MHC class II molecules (e.g., HLA-DRA) and CD74. They may originate from bone marrow-derived precursors under MIF-JAK/STAT3 signaling and influence T cell composition in the tumor microenvironment, potentially contributing to immune modulation and tumor progression. Emerging evidence suggests apCAFs may also derive from mesothelial cells via IL-1/TGF-β-mediated transdifferentiation. Notably, a S100A4⁺ apCAF subpopulation is associated with improved prognosis, indicating a possible tumor-restraining role. Metabolism-associated CAFs (meCAFs) express PLA2G2A and CRABP2 and exhibit elevated glycolytic activity. Although their enrichment is linked to increased metastatic potential, meCAFs are also associated with enhanced immune-cell infiltration. Vascular CAFs (vCAFs), which express IL-6 and CCL8, are primarily located in tumor cores and perivascular regions, where they interact with cancer cells via the IL-6/IL-6R axis. Tumor-derived exosomal miR-9-5p can further enhance IL-6 secretion by vCAFs.CD63⁺ CAFs mediate tamoxifen resistance through exosomal miR-22, regulated by SFRS1, with STAT3 activation maintaining their stable phenotype. Additionally, a rare PDGFRα^low^/CD34^hi^ Ptgs2⁺ fibroblast population constitutively secretes PGE₂ to activate the Ptger4-YAP axis, regulating stem-cell fate. Single-cell omics have also revealed numerous other specialized CAF subsets with context-specific regulatory functions.

However, despite the proliferation of single-cell datasets, many pan-tumor surveys remain primarily descriptive: they catalog transcriptional states and spatial patterns, but functional roles are frequently inferred rather than experimentally validated in vivo. Recent reviews synthesize a growing body of in vivo evidence that moves many CAF annotations from “marker–based inference” to experimental validation—for example, through subtype-specific ablation/depletion models, lineage-tracing/fate-mapping, and genetic or antibody-based perturbations that directly test ECM, immune and therapy-related functions [40]. A non-exhaustive overview of major CAF programs, their markers, and the strength of functional evidence is summarized in Table 1 and Fig. 1.

**Table 1:** Molecular features, functions, and cancer-specificity of CAF subtypes.

Subtype	Markers	Origin	Function/enriched pathway	Cancer specificity	Assay	Clinical significance
iCAF	CXCL1, IL6	Tissue-resident pancreatic stellate cells	Inflammation	Triple-negative breast cancer [41]	MARS-seq	Not reported
	ADH1B, GPX3, RGM, SCARA5, CCL19, CCL5	Not reported	Detoxification, response to stimuli, IFNγ, and cytokines	Breast cancer [58]	scRNA-seq	Not reported
	CXCL14, CCL2, SOCS3	Not reported	Not reported	Ovarian cancer [42]	scRNA-seq	Not reported
	PDGFRA, CXCL12, IL6, CXCL14, CXCL1, CXCL2	Not reported	ECM organization, regulation of cell migration, angiogenesis	Bladder carcinoma [12]	scRNA-seq	Promote tumor and stromal cell proliferation; recruit immune cells
	IL6, IL8, CXCL1, CXCL2, CCL2, CXCL12, CFD, LMNA, DPT	Not reported	IFNγ response, TNF/NF-κB, IL2/STAT5, IL6/JAK/STAT3, complement pathway	Pancreatic cancer (KPC mouse model) [36]	scRNA-seq	Not reported
	IL6, HAS1, COL14A1	Not reported	Not reported	Esophageal squamous cell carcinoma [61]	scRNA-seq	Not reported
myCAF	ATAC2, THBS2, FBN1, MFAP5	Tissue-resident pancreatic stellate cells	Wound healing	Triple-negative breast cancer [41]	MARS-seq	Not reported
	IGFBP3, FLT3, IRF7, GGH, PLP2, CST1, TGFβ, SEMA3C, SFRP4	Not reported	ECM, TGFβ signaling, wound healing, IFNα/β response, actomyosin	Breast cancer [58]	scRNA-seq	Participate in immunotherapy resistance
	POSTN, ACTA2, COMP, COL10A1, COL11A1, MMP11, TAGLN, FN1	Not reported	TGFβ signaling	Ovarian cancer [42]	scRNA-seq	Create dense matrix contributing to tumor cell exclusion
	RGS5	Not reported	Muscle system process, focal adhesion, ECM-associated pathways	Breast cancer [12]	scRNA-seq	Not reported
	TAGLN, MYL9, TPM1, TPM2, MMP11, POSTN, HOPX	Not reported	Smooth muscle contraction, focal adhesion, ECM organization, collagen formation	Pancreatic cancer (KPC mouse model) [36]	scRNA-seq	Not reported
	THBS2, COL12A1, THY1, IGKC, POSTN, COL1A1, CTHRC1, MMP1, MMP11	Not reported	Promote monocyte fibroblast proliferation	Esophageal squamous cell carcinoma [44]	scRNA-seq	Not reported
apCAF	H2-AB1, CD74, SLPI, PLA2G2A, CRABP2	Not reported	Antigen presentation	Triple-negative breast cancer [41]	MARS-seq	Not reported
	H2-AA, H2-AB1, SAA3, SLPI, COL1A1, COL1A2, DCN, PDPN	Not reported	Antigen processing, fatty-acid metabolism, MYC targets, mTORC1 signaling	Pancreatic cancer (KPC mouse model) [36]	scRNA-seq	Contribute to immune suppression in tumor microenvironment
	CD74, H2-AA, H2-AB1, H2-DMA, H2-DMB1, H2-EB1, MSLN, UPK3B, EZR, KRT19, NKAIN4, LRRN4	Mesothelial cells (IL-1 and TGFβ driven)	Induce naïve CD4^+^ T cells into regulatory T cells	Pancreatic cancer (KIC mouse model) [65]	scRNA-seq	Targeting Mesothelin may inhibit mesothelial-to-apCAF transition and immunosuppression
	CD74, HLA-DRA, HLA-DPB1	Not reported	Not reported	Esophageal squamous cell carcinoma [61]	scRNA-seq	Not reported
meCAF	PLA2G2A, CRABP2	Not reported	Translation, mitochondrial translation, glycolysis, noncanonical NF-κB, P21/P27 degradation, MYC pathways	Pancreatic ductal adenocarcinoma (loose-type) [68]	scRNA-seq	High abundance correlates with better immunotherapy response in PDAC patients
vCAF	NIDOGEN-2, NOTCH3, EPAS1, COL18A1, NR2F2, CSPG4, RGS5, PDGFRB, DES	Perivascular cells	Vascular development and angiogenesis	Breast cancer (MMTV-PyMT mouse model) [34]	scRNA-seq	Not reported
	CD146 (MCAM), MYH11, GJA4, RGS5, IL6, CCL8	Not reported	Muscle contraction, response to hypoxia, mesenchymal cell proliferation	Intrahepatic cholangiocarcinoma [62]	scRNA-seq	Not reported
CD63^+^ CAF	CD63, Col1a1, Col3a1, THY1, FAP	Not reported	TIMP1–CD63-Jak–STAT-STAT3 signaling, exosomal miR-22 induces drug resistance	Breast cancer (MMTV-PyMT mice) [70]	scRNA-seq	Therapeutic potential to enhance tamoxifen sensitivity in ERα-positive breast cancer
EMT-like CAFs	KRT19, KRT8, SAA1	Not reported	Epithelial-mesenchymal transition (EMT)	Intrahepatic cholangiocarcinoma [62]	scRNA-seq	Not reported
Lipo fibroblast	APOA2, FABP1, FABP4, FRZB	Not reported	Proliferation	Intrahepatic cholangiocarcinoma [62]	scRNA-seq	Not reported
TinCAF	CD40, CD276, NECTIN2, ENTPD1 (CD39), CD59, CAV1, MIF, NOTCH3	Not reported	Interferon response and oxidative phosphorylation	Colorectal cancer [20]	scRNA-seq + Spatial proteomics	Potential target for next-generation immune-checkpoint inhibitors
GlyCAF	SLC2A1 (GLUT1), PGK1, PKM, PGAM1, HK2	Not reported	Glycolysis and HIF-1 signaling	Soft-tissue sarcoma (mouse model) [142]	scRNA-seq	GLUT1 inhibitors may repolarize T cell-restrictive glyCAF
TSPAN8^*+*^ CAF	TSPAN8, GATAD1, MDM2, VEGFA	Not reported	Histone modification and chromatin remodeling	Pan-cancer [84]	scRNA-seq + ST	Not reported

*CAF* cancer-associated fibroblast, *iCAF* inflammatory cancer-associated fibroblast, *myCAF* myofibroblastic cancer-associated fibroblast, *apCAF* antigen-presenting cancer-associated fibroblast, *meCAF* metabolic cancer-associated fibroblast, *vCAF* vascular cancer-associated fibroblast, *TinCAF* T cell-influencing cancer-associated fibroblast, *EMT* epithelial-mesenchymal transition, *ECM* extracellular matrix, *scRNA-seq* single-Cell RNA sequencing, *ST* spatial transcriptomics, *MARS-seq* massively parallel RNA single-cell sequencing, *MMTV-PyMT* mouse mammary tumor virus-polyoma middle T antigen, *KPC* Kras^LSL-G12D/+^; Trp53^flox/flox^; Pdx1-Cre; KIC, Kras^LSL-G12D/+^; Ink4a^fl/fl^; Ptf1a^Cre/+^.

### Inflammatory CAFs (iCAFs): a central hub for cytokine signaling, immune reprogramming, and therapy resistance

Inflammatory CAFs (iCAFs) represent a major regulatory fibroblast program uncovered by single-cell and spatial multi-omics. In PDAC, iCAFs occupy stromal regions distinct from tumor nests [32, 36], express low ACTA2, and secrete high levels of cytokines including IL-6, CXCL12, and CCL2 [41–44]. Their differentiation is jointly governed by IL1R1/JAK/STAT and NF-κB signaling, relationships demonstrated through perturbation assays in organoid and co-culture systems [45]. Functionally, iCAFs reinforce an immunosuppressive milieu through two major routes [32]: (i) IL-6 and CXCL12 secretion suppresses CD8⁺ T cell cytotoxicity, accompanied by PD-1 upregulation observed in MEL scRNA-seq; (ii) HGF–MET activation promotes intraductal papillary mucinous neoplasm (IPMN) growth, establishing trophic support for malignant epithelium [46–48], several primary studies now use these complementary approaches to confirm cytokine-driven iCAF effects on T cell function and therapeutic resistance [40].

Across tumor types, iCAFs exhibit context-specific functional polarization. In gastric adenocarcinoma, a transcriptionally defined IFNγ⁻ iCAF state facilitates immune escape [49]. In BlCa, COL1A1⁺PDGFRA⁺ iCAFs are enriched for ECM remodeling and inflammatory programs, correlating with advanced disease stage [12]. In BC, iCAFs contribute to anti-PD-1 resistance by promoting tumor proliferation, driving EMT, and reinforcing an immunosuppressive niche [35]. Late–stage cutaneous malignancies similarly show iCAF accumulation with chemokine–rich profiles that coordinate immune-cell recruitment [50]. A recently proposed pan-cancer iCAF score integrates these programs to predict prognosis and immunotherapy benefit, underscoring their translational potential [51] (Table 1).

Single-cell profiling in human PDAC has also identified complement-associated CAFs (csCAFs) enriched for C3 and C7 transcripts [52]. Although their function was not directly validated, their association with inflammatory pathways and the dual role of complement in tumor progression [53], suggest a context-dependent contribution to PDAC-related immune modulation. Recent CRISPR Perturb-seq models further demonstrate that iCAF identity is defined by distinct regulatory dependencies. Targeted disruption of pathways such as TGFBR1, AEBP1, PTGS1, and IL1R1-STAT3 produced subtype-specific transcriptional and functional shifts, altering IL-6 secretion, ECM remodeling, and interferon responsiveness in ways that aligned with iCAF–like versus myCAF-like phenotypes. These experiments provide direct functional validation for the cytokine-driven regulatory architecture of iCAFs, supporting their delineation as a biologically coherent and experimentally tractable fibroblast program [54] (Table 1).

### MyCAF: the mechanical axis of matrix remodeling and immune resistance

Myofibroblastic CAFs (myCAFs) constitute a contractile, ECM remodeling stromal population consistently identified across species. Integrated scRNA-seq analyses from human tumors and murine PDAC (KPC model) show that myCAFs localize immediately adjacent to tumor cells [32, 36], express high levels of ACTA2 and contractility genes (*TAGLN, MYL9*, and *TPM1*/*TPM2*), and are enriched for ECM-modifying factors such as MMP11 and POSTN, alongside transcriptional regulators including HOPX [35]. These features equip myCAFs to mediate collagen deposition, mechanical-stress propagation, and matrix reorganization. Consistent with these predicted functions, myCAFs were experimentally shown to promote cancer-cell invasion in a transwell Matrigel assay, where they significantly increased the number of malignant cells traversing the matrix [34].

Transition to the myCAF phenotype is principally driven by TGF-β signaling [55]. Biffi et al. demonstrated that TGF-β–dependent SMAD activation converts normal fibroblasts into myCAFs [45], and in gastric adenocarcinoma, TGF-β2/β3 isoforms promote ECM stabilization and tumor progression [55]. In PDAC, LRRC15⁺ TGF-β–responsive myCAFs emerge as a dominant late-stage stromal population, and functional perturbation studies show that LRRC15⁺ enrichment contributes to poor response to anti-PD-L1 therapy, as evidenced by fibroblast-targeting and TGF-β blockade experiments [56]. In BC, CD26⁺ normal fibroblasts similarly give rise to myCAFs [57].

CAF heterogeneity within the myCAF lineage is further illustrated by multi-marker studies. A BC framework defining CAF-S1–S4 based on six fibroblast markers was later refined by scRNA-seq [35], which subdivided CAF-S1 into eight clusters, including ECM-myCAF, TGFβ-myCAF, and wound-myCAF—three subpopulations enriched in PD-1–resistant microenvironments of NSCLC and HNSCC [58]. Notably, ECM-myCAFs spatially associate with immunosuppressive cell populations such as TREM2⁺ macrophages, regulatory NK cells, and Tregs [59].

A specialized senescent myCAF (senCAF) subset has also been identified in BC, characterized by ECM-driven suppression of NK cytotoxicity and promotion of recurrence across HER2⁺, ER⁺, and triple-negative subtypes [59, 60]. Senolytic targeting of senCAFs delays tumor progression, underscoring their translational relevance. Together, these findings expand the mechanistic landscape of myCAF biology and highlight therapeutic opportunities focused on LRRC15⁺ myCAFs and senCAFs (Table 1).

### ApCAF: a functional axis bridging antigen presentation and immune modulation

Antigen-presenting CAFs (apCAFs) constitute a distinct fibroblast program identified by single-cell transcriptomics [36]. Unlike myCAFs and iCAFs, apCAFs exhibit prominent expression of MHC class II genes (*HLA-DRA* and *HLA-DPB1*) together with the invariant chain CD74 [61–63], suggesting an intrinsic potential for antigen presentation. Functional studies indicate that apCAFs may arise from bone marrow-derived precursors exposed to macrophage migration inhibitory factor (MIF), which activates JAK–STAT3 signaling and reshapes local immunity. In HNSCC, this lineage skews the intratumoral CD4⁺/CD8⁺ T cell ratio and promotes disease progression [64]. Metabolic pathway analyses further reveal apCAF enrichment for fatty-acid metabolism, mTORC1 signaling, MYC targets, and antigen-processing programs [36], highlighting their integrated metabolic–immunologic reprogramming.

Single-cell re-analyses also support a mesothelial origin for apCAFs: IL-1 and TGF-β signaling downregulate mesothelial markers while inducing fibroblastic programs such as ACTA2 [65], and apCAFs frequently co-cluster with mesothelial cells across cancers [56]. Context–dependent roles have been reported. In TNBC models, apCAFs comprise a subset of S100A4⁺ sCAFs; a high S100A4⁺ apCAF:PDPN⁺ pCAF ratio aligns with BRCA mutations and better prognosis, suggesting a tumor-restraining function in specific genetic backgrounds [41]. Conversely, in HCC, apCAF activity remains incompletely defined and may interface with lipid-metabolic CAF states (lpCAFs) [66]. ApCAF-like cells are also found in healthy mouse tissues, including the mammary gland and pancreas, implying roles in physiological stromal homeostasis [67].

A central question concerns their functional duality: although apCAFs frequently reinforce immunosuppression, in select contexts—such as BRCA-mutant breast cancer—they may limit tumor growth. This dichotomy likely reflects microenvironment–specific interactions with distinct epithelial and immune compartments (Table 1).

### Functionally specialized CAF subtypes: hubs for metabolic, vascular, and inflammatory crosstalk

#### Metabolism-associated CAFs (meCAFs)

Single-cell profiling of PDAC with loose stroma identified a metabolism-associated CAF population (meCAFs) characterized by high PLA2G2A and CRABP2 expression and enrichment of glycolytic and mitochondrial translation–elongation pathways [68]. By engaging in compartmentalized metabolic programming—vigorous glycolysis in meCAFs contrasted with oxidative phosphorylation in adjacent tumor cells—these fibroblasts establish a metabolic symbiosis network. meCAFs also interact more extensively with macrophages than myCAFs or iCAFs, highlighting their immunometabolic connectivity. Although meCAF-rich regions correlate with metastatic potential, they paradoxically exhibit higher CD8⁺ T cell infiltration and may predict improved immunotherapy responsiveness. Collectively, these findings position meCAFs as metabolic hubs and potential biomarkers of therapeutic response in PDAC (Table 1).

#### Vascular CAFs (vCAFs)

In BC, single-cell analyses reveal a proangiogenic CAF subset—vascular CAFs (vCAFs)—that transcriptionally mirrors endothelial programs during early vascular remodeling [49]. In intrahepatic cholangiocarcinoma (ICC), CD146⁺ vCAFs secrete IL-6 and CCL8 and adopt endothelial-like signatures [62]. Spatial mapping places vCAFs in perivascular niches where they communicate with tumor cells through an IL-6/IL-6R signaling loop, a vessel-centric dialog validated in murine ICC models [69]. Tumor-derived exosomal miR-9-5p enhances IL-6 release by vCAFs, and vCAF-derived IL-6 increases EZH2 in ICC cells, reinforcing malignancy. Additional EMT-like and lipid-metabolic CAF programs (KRT19⁺/KRT8⁺; FABP1⁺/APOA2⁺) co-exist within this stromal ecosystem [69] (Table 1).

#### CD63^+^ CAFs

In late-stage breast cancer, CD63⁺ CAFs drive tamoxifen resistance via SFRS1-controlled exosomal miR-22, which suppresses ERα and PTEN and maintains STAT3 activation [70]. Neutralizing CD63 with antibodies or cRGD–miR-22–sponge nanoparticles restores drug sensitivity. Similar CD63⁺ fibroblast subsets have been identified in PCa CAF cultures [71], suggesting broader therapeutic relevance across tumor types (Table 1).

#### Pericryptic Ptgs2^+^ fibroblasts

A rare Ptgs2⁺ fibroblast population (PDGFRαˡᵒʷ/CD34ʰⁱᵍʰ) in the murine intestinal mesenchyme continuously secretes PGE₂, activating a Ptger4YAP circuit that controls stem-cell expansion and tumor initiation [72]. Ptger4 deletion blocks Sca-1⁺ stem-cell proliferation, while COX-2 inhibition reduces tumorigenesis in Apc^min/+^ mice. Analogous fibroblasts reside at the base of normal human colonic crypts [73], linking this lineage to epithelial homeostasis and early tumorigenesis (Table 1).

#### Interferon-responsive CAFs (ifCAFs)

In human PDAC, scRNA-seq of FAP⁺ mesenchymal cells identifies an interferon-responsive CAF subset (ifCAFs) enriched for IFN-stimulated genes and capable of restraining tumor growth in co-culture assays [74]. In murine breast cancer, TGF-β blockade induces interferon-licensed fibroblasts (ilCAFs) that enhance immune-cell recruitment and modulate inflammatory cues [75]. These studies establish IFN-responsive CAFs as a conserved, functionally validated immune-modulatory fibroblast program across tumor types (Table 1)

### Tumor-specific CAFs: key mediators of stem-cell niches and tissue-specific regulation

Tumor-specific CAF programs often act as niche organizers for cancer stem cells and as tissue-adapted regulators of matrix and immune dynamics. In BC, a CD10⁺GPR77⁺ CAF subset sustains cancer stemness and chemoresistance by maintaining NF-κB signaling and secreting IL-6 and IL-8; neutralization of GPR77 suppresses tumor growth and restores chemosensitivity in xenograft models [76]. In early-stage bladder cancer, PDGFRα⁺ITGA11⁺ CAFs promote lymphovascular invasion via SELE–ITGA11 engagement and SRC–VEGFR3–MAPK activation, while secreting CHI3L1 to remodel the peritumoral matrix and enhance metastasis [77]. In CRC, integrative single-cell and spatial analyses identify a T cell–inhibitory CAF subset (TinCAFs) enriched for CD40 and NECTIN2; NECTIN2 blockade reverses TinCAF-mediated suppression of effector T cell proliferation in vivo, establishing their immunoregulatory function [20]. Together, these examples highlight CD10⁺GPR77⁺ CAFs, PDGFRα⁺ ITGA11⁺ CAFs, and TinCAFs as mechanistically validated tumor-specific stromal drivers of progression.

Additional tissue-restricted CAF states further illustrate the breadth of functional specialization. In HNSCC, an elastic-fiber–remodeling ELN⁺/FBLN1⁺/MFAP4⁺ CAF population associates with a favorable prognosis in HPV⁺ disease, suggesting a potential immune-modulatory role [78], whereas in mesothelioma, “Meso-CAFs” expressing ELN and TCF21 enhance tumor-cell proliferation and migration through c-Met/PI3K and WNT signaling [79]. In liver cancers, vascular (CD146⁺), antigen-presenting (MHC-II⁺), epithelial-like, mesothelial-like, and lipid-processing CAFs (APOA1/2⁺, APOC1⁺ and CD36⁺ myofibroblastic counterparts) occupy discrete niches and support tumor growth through validated cholesterol handling and ECM-remodeling activities; spatial transcriptomics links these “onco-fetal” neighborhoods to relapse and immunotherapy response [80]. In NSCLC, three CAF programs (HGF⁺FGF7⁺, HGF⁻FGF7⁺, and HGF⁻FGF7⁻) show distinct functional dependencies, with combined MET and FGFR inhibition required to suppress HGF⁺FGF7⁺ CAF-driven signaling [81]. Collectively, these mechanistic studies move beyond marker-based taxonomies and establish tissue-specific CAF subsets as active determinants of tumor progression (Table 1 and Fig. 1).

### Pan-cancer CAF lineages: cross-tissue integration and subtype convergence

To chart the broader landscape of CAF states across malignancies, several integrative efforts have constructed pan-cancer fibroblast atlases using scRNA-seq and bulk transcriptomics. Buechler et al. merged 50 scRNA-seq datasets and identified two “core” fibroblast lineages conserved across 17 healthy and diseased murine tissues, providing a baseline for cross-context fibroblast comparisons. Galbo et al. extended this view by analysing bulk RNA-seq from 31 tumor types and defining six pan-CAF programs, including collagen-remodeling Pan-dCAFs, homeostatic Pan-nCAFs, and proliferation-linked Pan-pCAFs [82]. Luo et al. harmonized 12 single-cell datasets from ten solid tumors and resolved two normal-fibroblast and six CAF states; three dominant functional modules—CAFmyo (contractile), CAFinfla (inflammatory), and CAFadi (adipogenic/lipid-metabolic)—were shared across cancers, while CAF_state2_ (PDGFRB⁺, angiogenic) and CAF_state3_ (FAP⁺, immune-modulatory) showed context-dependent shifts, underscoring microenvironment-specific regulation of CAF behavior [83].

Further expanding this landscape, Liu et al. assembled a meta-atlas of 249,156 single-cell transcriptomes from 73 studies across ten tissues and defined 18 shared fibroblast subtypes [84]. Among these, a previously uncharacterized TSPAN8⁺ fibroblast population expressed TSPAN8, GATAD1, MDM2, and VEGFA and was enriched for chromatin remodeling and histone-modification pathways, with ligand–receptor modeling suggesting VEGFA–F2R-mediated communication with endothelial and T cells and correlations with poor clinical outcome. However, these observations remain largely transcriptomic: direct experimental validation of chromatin remodeling or paracrine functions is still lacking, and cross-tissue conservation has so far been confirmed in only a subset of single-cell datasets from pancreas, colon, and lung [84]. Together, these atlases reveal a convergent set of CAF modules (CAFmyo, CAFinfla, CAFadi, and related angiogenic/immune-regulatory states) but also highlight the current gap between descriptive lineage maps and mechanistically proven functions.

### Functional duality and plasticity of CAFs: dynamic balance between pro- and antitumor states

CAFs exhibit pronounced phenotypic and functional duality and are broadly categorized into pro-tumorigenic CAFs (pCAFs) and tumor-restraining CAFs (rCAFs) [85]. While pCAFs predominate in most TMEs, rCAFs can counteract tumor growth through discrete molecular mechanisms [86]. In PDAC and CRC, for example, myCAF-derived collagen I can impose mechanical constraints on tumor expansion [46]. In PDAC, a meflin⁺ CAF subset suppresses cancer stemness via BMP4 secretion [87]. In CRC, multi-omics profiling has identified a terminally differentiated, mucosa-derived fibroblast population marked by ADAMDEC1, CXCL14, EDNRB, and PROCR, enriched for ECM-attenuating and immune-activating programs and associated with favorable prognosis and improved immunotherapy response [88]. Epigenomic data suggest that rCAF identity is stabilized by super-enhancer–driven networks rather than transient transcriptional fluctuations, and ADAMDEC1 expression in CAFs correlates with T cell infiltration, although a direct causal link to immune activation remains to be demonstrated [88].

Recent work has begun to map the spatial and temporal organization of CAF states. In pancreatic cancer, CD105⁺ and CD105⁻ fibroblasts represent genetically and functionally distinct lineages that do not interconvert: CD105⁺ CAFs exhibit heightened TGF-β responsiveness and tumor-promoting activity, whereas CD105⁻ CAFs display tumor-restraining features and preferentially express antigen-presenting markers such as MHC-II and CD74 [89]. Parallel lineage-tracing and single-cell studies reveal a plastic axis between inflammatory iCAFs and myofibroblastic myCAFs: iCAFs can transition toward myCAFs upon TGF-β signaling or IL-1/JAK/STAT inhibition, passing through a transient α-SMA⁺/p-STAT3⁺ intermediate state [32, 67]. Therapeutic pressures further reshape these trajectories; for instance, androgen-receptor blockade enhances TGF-β activity and drives stromal reprogramming toward an SPP1⁺ myCAF-like phenotype [90], while myCAFs can partially revert toward iCAF-like states under altered cytokine conditions. Conceptual advances also emphasize that CAF heterogeneity reflects both stable lineage programs and dynamic, therapy-responsive state transitions. A recent synthesis of stromal biology highlights that CAF states differ not only transcriptionally but also in temporal kinetics, turnover rates, and niche residency. For example, TGF-β–driven myCAF states typically emerge in late, mechanically stiff tumor regions, whereas inflammatory iCAFs preferentially arise under early or cytokine-rich conditions. These temporally and spatially constrained stromal programs influence tumor evolution and therapeutic sensitivity, supporting a framework in which CAF diversity represents a continuum shaped by microenvironmental pressures rather than static clusters [40].

## TAM diversity: dissecting lineages and dynamic functional states

### The limitations of the M1/M2 polarization model and the dynamic complexity of TAM functions in the TME

The classical M1/M2 polarization model—derived largely from in vitro studies—divides macrophages into pro-inflammatory (M1) and immunosuppressive (M2) phenotypes. M1-like macrophages are classically activated by IFN-γ or LPS and exert antitumor effects through pro-inflammatory cytokines and cytotoxic mediators such as ROS and TNF [91]. In contrast, M2-like macrophages arise under alternative activation by IL-4 and IL-13 and support angiogenesis, tissue repair, and immune suppression by secreting factors including VEGF and MMPs, thereby promoting cancer-cell invasion and ECM remodeling [91–93].

However, this binary framework is overly simplistic in the context of the TME. Accumulating evidence indicates that TAMs display pronounced plasticity and tissue specificity, with functional states sculpted by hypoxia, metabolic by-products, and complex cytokine networks. TAMs frequently co-express markers traditionally viewed as mutually exclusive—for example, PD-L1⁺/Arg1⁺—and their transcriptional programs vary markedly across tumor types [94]. The advent of single-cell and spatial multi-omics, including scRNA-seq, scATAC-seq, and spatial metabolomics, has therefore moved beyond the M1/M2 dichotomy, revealing a continuum of TAM states and profound intratumoral heterogeneity.

Recent integrative reviews emphasize that transcriptomic diversity must be interpreted together with in vivo functional evidence. The fifth article synthesizes depletion, lineage-tracing, and perturbation studies showing that key TAM states—such as SPP1⁺, C1Q⁺, TREM2⁺, and metabolic TAMs—exert causal effects on angiogenesis, immune suppression, metastatic conditioning, and therapeutic resistance. This framework highlights that TAM heterogeneity is not only transcriptional but also spatiotemporal and functionally validated, and that discrete TAM subsets can be mechanistically linked to treatment response [95].

#### SPP1^+^ TAMs

SPP1⁺ TAMs have emerged as a reproducibly observed macrophage state across CRC, LUAD, BC, and EC, marked by high expression of SPP1, FN1, IL1RN, and M2-like markers such as CD206, PD-L1, and CD163. Multiple scRNA-seq studies consistently report enrichment of angiogenic, ECM–receptor–interaction, and wound-healing programs in this population, suggesting potential involvement in angiogenesis and matrix remodeling [96–100]. They are characterized by high expression of SPP1, FN1, and IL1RN together with canonical M2-like markers such as CD206, PD-L1, and CD163, and single-cell transcriptomic analyses consistently show enrichment of angiogenic and ECM–receptor–interaction programs [96, 101, 102]. Likewise, recent work using spatial and single-cell profiling coupled with perturbational assays has shown that SPP1-expressing macrophages can promote local ECM changes and impair CD8⁺ T cell effector function in metastatic niches, and that blocking SPP1–receptor interactions (for example, SPP1–CD44) can reverse aspects of T cell dysfunction in model systems [103]. Together, these data establish SPP1⁺ TAMs as a prototypical protumor subset integrating angiogenic, matrix-remodeling, and immune-modulatory functions. Importantly, a growing but still limited set of interventional studies now provides direct functional evidence implicating the osteopontin (OPN/SPP1) axis in protumor macrophage biology. In preclinical BC models, macrophage depletion or pharmacologic/genetic inhibition of OPN reduced metastatic outgrowth, restored T cell infiltration and improved responses to anti-PD-1 therapy, supporting a causal role for OPN in driving macrophage-dependent immune suppression and metastasis [104]. SPP1⁺ macrophages represent one of the most functionally validated TAM programs to date. Genetic and pharmacologic inhibition of the SPP1–CD44 axis, macrophage depletion strategies support a causal role for SPP1⁺ TAMs in resistance to PD-1/PD-L1 blockade [95].

A transcriptionally related macrophage population expressing CCL18 has been described in BC, NSCLC, CRC, and HCC [105, 106]. CCL18⁺ TAMs share M2-like immunosuppressive features with SPP1⁺ cells but show an even stronger association with tumor progression and poor prognosis [105]. In NSCLC, CCL18⁺ TAMs are metabolically biased toward fatty-acid oxidative phosphorylation (FAO), whereas SPP1⁺ TAMs rely more on glycolysis to fuel angiogenesis and metastatic dissemination [105], linking subset-specific metabolic wiring to divergent protumor functions. In HNSCC, a higher CXCL9/SPP1 ratio correlates with increased T- and B-cell infiltration, highlighting its potential as a biomarker of immune microenvironment activity [107]. Although most evidence for CCL18⁺ TAMs remains correlative and primarily transcriptomic, their close similarity to SPP1⁺ TAMs suggests that they likely represent a highly protumor branch within the broader SPP1⁺ macrophage continuum, warranting further functional dissection [105, 106].

#### C1Q^+^ TAMs

C1Q⁺ TAMs are characterized by robust expression of complement components (C1QA, C1QB, and C1QC) together with immunoregulatory molecules such as TREM2, MERTK, PD-L1, and CD80 [96, 108, 109]. This subset has been reported across multiple tumor entities, including CRC [96], NSCLC [102], BC [110], pancreatic cancer (PCA) [111], HCC [112], and cholangiocarcinoma (CC) [113]. Single-cell and spatial transcriptomic analyses show that C1Q⁺APOE⁺ TAMs in CRC and renal-cell carcinoma adopt an immunosuppressive, phagocytosis-competent phenotype, enriched for M2-like and engulfment programs, positioned near exhausted T cells, and expressing high levels of PD-L1 and MERTK [96, 109, 114, 115], although the majority of data remain correlative. Intriguingly, in MEL and LUAD, C1Q⁺ TAM infiltration has been associated with favorable responses to immune-checkpoint blockade [116–118], and in CC, abundant C1QC⁺ TAMs correlate with improved prognosis [113]. Thus, in addition to their immunosuppressive and phagocytic activity [96], C1Q⁺ TAMs exhibit context-dependent associations with outcome, underscoring their multifaceted contributions to tumor immunity (Table 2).

**Table 2:** Molecular markers, metabolic features, and functions of TAM subtypes.

Subtype	Markers	Origin	Function/enriched pathway	Cancer distribution	Assay
SPP1^+^ TAM	SPP1, MARCO, VEGFA	Tumor-infiltrating monocyte-like precursors, RTMs	Angiogenesis, ECM–receptor interaction, tumor vasculature pathways, tumor growth and metastasis	Colorectal cancer [95]	scRNA-seq
	SPP1, MMP9, CSTB, GAPDH, MT2A TF: *FOSL2*, *CEBPB*	Not reported	Angiogenesis, ECM proteolysis, tumor-associated fibroblasts, glycolysis	Non-small cell lung cancer [91]	scRNA-seq
	SPP1, APOC1, APOE, TREM2, FN1, LGALS3, FTL, CD9, CTSB	Not reported	Glycosphingolipid metabolism, glucose metabolism, HDL-mediated lipid transport, and construction of collagenous ECM	Metastatic colorectal cancer [103]	scRNA-seq
	SPP1, CSTB, SDC2	Not reported	Glycolysis, EMT, angiogenesis, hypoxia	Colorectal cancer [101]	scRNA-seq
	SPP1, HTRA1, C3, OLFML3, APOC1, CD9, CCL3L1	Not reported	Increased oxidative phosphorylation, glycine/serine/threonine/tyrosine metabolism	Intrahepatic cholangiocarcinoma [106]	scRNA-seq
	SPP1, CD68	Not reported	Neutrophil activation, ATP metabolic process, oxidative phosphorylation, respiratory electron transport chain	Head and neck squamous cell carcinoma [110]	scRNA-seq
CCL18^+^ TAM	CCL18, FTL, APOC1, APOE, C1QB TF: *NR1H3*	Not reported	Fatty-acid oxidative phosphorylation, PPARG signaling, canonical M2 pathways, complement cascade, IFNγ response, antigen presentation	Non-small cell lung cancer [105]	scRNA-seq
	CCL13, CCL18, MRC1, CD209, CD163, MARCO, CSF1R	Not reported	Nitrogen and riboflavin metabolism	Intrahepatic cholangiocarcinoma [106]	scRNA-seq
C1Q^+^ TAM	C1QA/C1QB/C1QC, TREM2, MERTK, CD80	Tumor-infiltrating monocyte-like precursors	Complement activation, antigen processing/presentation, T cell recruitment/activation	Colorectal cancer [95]	scRNA-seq
	C1QA, C1QB, C1QC, CXCL10, HLA-DRB1, ISG15 TF: *IRF1*, *IRF7*, *STAT1*	Not reported	Immune-promoting functions	Non-small cell lung cancer [105]	scRNA-seq
	C1QA, C1QB, C1QC	Not reported	Antigen presentation, pathogen phagocytosis	Colorectal cancer [101]	scRNA-seq
FCN1^+^ TAM	FCN1, FOS, TIMP1, S100A8, S100A9, VCAN TF: *REL*, *XBP1*	Not reported	Pro-inflammatory	Non-small cell lung cancer [105	scRNA-seq
	FCN1, S100A8, S100A9	Not reported	Angiogenesis	Colorectal cancer [101]	scRNA-seq
LA-TAMs	TREM2, LGALS3, APOE, SYNGR1, CSTB, CD63, FABP5, TIMP2, CTSL	Not reported	Lysosome function, cholesterol efflux, lipid catabolism, EMT, T cell regulation, endothelial proliferation, ECM pathways	Breast cancer (mouse) [127]	scRNA-seq
LAM1/2	TREM2, FABP5, APOE, CCL18	Not reported	Immunoregulation	Breast cancer [129]	scRNA-seq + CITE-seq
Angio-TAM	F4/80, CD115, C3aR, CD88	F4/80^+^ HO-1^+^ bone marrow precursors	Heme catabolism (HO-1), immunosuppression, angiogenesis, EMT	Melanoma [132]	scRNA-seq
TAM_CD44	CD44, VEGFA, FOSL2, TIMP2, TEAD1, RUNX3	CD14^+^ monocytes and CX3CR1^+^ TAMs	Tumor angiogenesis	Ependymoma [133]	scRNA-seq, scATAC-seq, ChIP-seq

*TAM* tumor-associated macrophage, *LAM* lipid-associated macrophage, EMT epithelial-mesenchymal transition, *ECM* extracellular matrix, *scRNA-seq* single-cell RNA sequencing, *ST* spatial transcriptomics, *ChIP-seq* chromatin immunoprecipitation sequencing, *CITE-seq* cellular indexing of transcriptomes and epitopes by sequencing, *scATAC-seq* single-cell assay for transposase-accessible chromatin with sequencing, *RTM* resident tissue macrophage, *TF* transcription factor.

A related FCN1⁺ TAM population is thought to represent a developmental intermediate within the C1Q⁺ lineage [96, 101, 112]. Enriched in tumor-adjacent tissues and detected in NSCLC [105], HCC [112], and PCA [111], FCN1⁺ TAMs express inflammatory genes (e.g., *FCN1, FYN*, and *STAT1*) and remodel the microenvironment via proangiogenic activity. Lineage analyses suggest that FCN1⁺ TAMs can differentiate into both C1Q⁺ and SPP1⁺ TAMs [96, 101], positioning them as a transitional node within TAM trajectories and a potential therapeutic entry point for redirecting macrophage fate in tumors.

#### Lipid-associated macrophages (LAMs)

Lipid-associated macrophages (LAMs) are defined by high expression of lipid-metabolism genes such as APOC1, APOE, and FABP5 and are widely observed in GC, CRC, HCC, and pancreatic cancer [94, 96, 110, 119–121]. Functionally, LA-TAMs are centered on cholesterol metabolism and fatty-acid oxidation. In CRC and matched liver metastases, MRC1⁺ TAMs with LAM-like metabolic signatures co-localize with CD47⁺ tumor cells; spatial profiling shows enrichment of cholesterol metabolism, FAO, and complement pathways, and these cells are markedly enriched in chemotherapy-resistant specimens, suggesting utility as prognostic markers and potential CD47-directed therapeutic targets [122].

A conserved TREM2⁺ LAM subset was first described by time-resolved scRNA-seq in obese adipose tissue [123] and later identified in pre- and post-treatment samples from patients with advanced NSCLC [124]. Across NSCLC and BC, higher TREM2⁺ macrophage abundance inversely correlates with cytotoxic CD8⁺ T cell infiltration and response to PD-1/PD-L1 blockade [124–126]. In murine models of breast cancer metastasis, Trem2⁺Lgals3⁺ TAMs display defective phagocytosis and secrete profibrotic mediators that remodel ECM and reinforce immune suppression [127], while genetic or therapeutic depletion of TREM2 reprograms macrophage metabolism and restores antitumor T cell activity, functionally validating its protumor role [128]. In TNBC, scRNA-seq studies show that FAP⁺ CAFs drive monocyte differentiation into STAB1⁺TREM2⁺ LAMs via the CXCL12–CXCR4 axis; these LAMs suppress T cell activation and proliferation, are enriched in ICB-resistant tumors, and their depletion curtails tumor growth [13].

Using CITE-seq and spatial transcriptomics, Garrido et al. further resolved two TREM2-dependent LAM subsets: LAM1 (FABP5⁺), whose infiltration correlates with poor survival, and LAM2 (APOE⁺), which promotes immunosuppression via APOE-mediated cholesterol efflux in PD-1/PD-L1⁺ microenvironments; both subsets require TREM2 for lipid homeostasis, and TREM2 deficiency disrupts their metabolic programming [129]. Pan-cancer analyses confirm accumulation of TREM2⁺ macrophages in lung, colon, liver, breast, gastric, and pancreatic tumors, and indicate a monocytic origin; across ten tumor types, five TREM2⁺ TAM clusters—including PDGFB⁺ and FOLR2⁺ subsets—compose ~30% of the macrophage compartment and associate with exhausted CD8⁺ T cells [94, 128]. Notably, SPP1 is frequently reported as a marker gene for TREM2⁺ macrophages, and TREM2⁺ clusters closely align with SPP1⁺ macrophage populations in colon cancer, suggesting substantial overlap between SPP1⁺ and TREM2⁺ TAM states [94, 130] (Table 2).

#### Angiogenesis-associated TAMs (angio-TAMs)

Angiogenesis-associated TAMs (angio-TAMs) constitute a macrophage state transcriptionally enriched for hypoxia- and vessel-related programs. In NSCLC, PDAC, and ovarian cancer, scRNA-seq identifies HIF1A⁺SLC2A1⁺ macrophages localized to hypoxic regions of the TME, suggesting a role in tumor adaptation to low oxygen and support of neovascularization [105, 111, 131]. Functional evidence comes primarily from murine models: in fibrosarcoma and MEL, macrophage-specific HO-1 activity drives epithelial-mesenchymal transition, angiogenesis, and the formation of metastasis-permissive niches [132], demonstrating that heme-degradation–mediated signaling in TAMs can facilitate tumor dissemination. In human spinal ependymoma and murine glioma, scATAC-seq further delineates an Angio-TAM (CD44⁺VEGFA⁺) population with accessible chromatin enriched for TEAD1 and CEBP motifs [133], pointing to candidate transcriptional regulators of hypoxia- and vasculature-associated programs, though mechanistic confirmation remains limited.

A related LYVE1⁺ perivascular TAM (PvTAM) subset has been described in invasive BC and MMTV-PyMT mouse models [134]. These LYVE1⁺ macrophages cluster around αSMA⁺ pericyte-like stromal cells to form angiogenic niches organized by PDGFRα–PDGF-CC signaling, while spatial transcriptomics implicates IL-6–induced CCR5 expression as a key cue for their perivascular positioning [135]. HO-1 activity in LYVE1⁺ PvTAMs further contributes to exclusion of CD8⁺ T cells from the TME, linking vascular remodeling to immune evasion [135]. Together, angio-TAMs and LYVE1⁺ PvTAMs define a transcriptionally and spatially distinct proangiogenic TAM axis that couples vascular remodeling with local immunosuppression (Table 2).

#### Other TAM subsets

Pan-cancer analyses highlight metabolic heterogeneity as a key determinant of TAM function and the immune landscape. One macrophage subset present across multiple tumor types expresses high levels of heat-shock proteins (HSPs), suggesting a role in stress-response pathways and tumor progression, although mechanistic data remain sparse [94]. More concrete evidence comes from metabolically rewired TAMs in CRC, where the glycolytic enzyme PFKFB3 marks an immunosuppressive macrophage population. Spatial multi-omics with NanoString GeoMx shows that PFKFB3-high TAMs co-localize with CD163⁺ M2-like macrophages in glycolytic, immunosuppressive regions [136]. Functionally, monocytes from patients with colon (but not rectal) cancer overexpress PFKFB3, adopt a pro-tumoral phenotype, and promote macrophage-mediated immune suppression in vitro, and elevated PFKFB3 mRNA levels correlate with shorter overall and progression-free survival [136, 137]. These findings position PFKFB3 as both a biomarker and a metabolic regulator of immunosuppressive TAM states in CRC.

Checkpoint-enriched TAMs provide another layer of immune regulation. In oral squamous cell carcinoma (OSSC) and premalignant lesions, a TAM subset termed Macro-IDO1 overexpresses IDO1 and drives T cell exhaustion through the IFN-γ–JAK–STAT axis. Spatial transcriptomics reveals that Macro-IDO1 TAMs form immunosuppressive hotspots at tumor margins by co-localizing with PD-1⁺TIM-3⁺ exhausted T cells. Pharmacologic IDO1 inhibition restores T cell function and restrains tumor growth in preclinical models [138], illustrating that metabolic enzymes and immune checkpoints can be spatially co-regulated within TAM niches and constitute actionable targets for reversing immune escape.

**Fig. 2:**
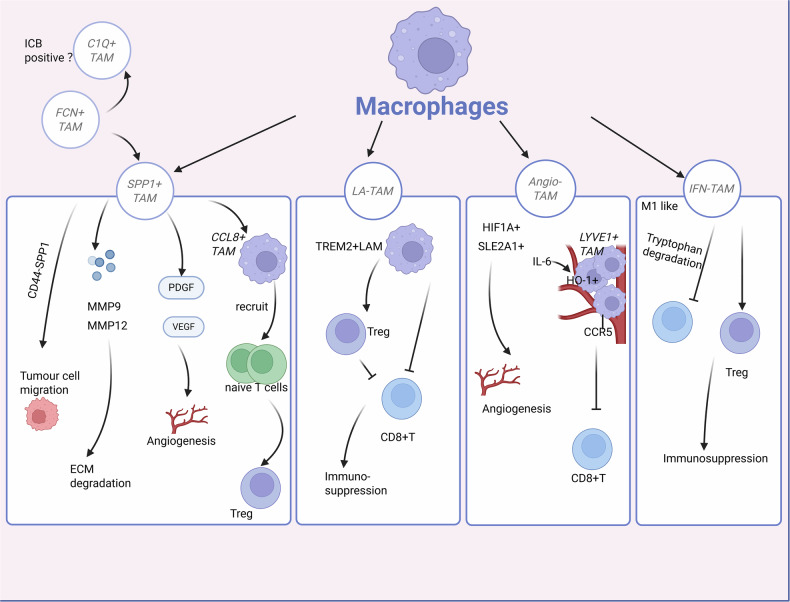
Phenotypic diversity and functional specialization of tumor-associated macrophage (TAM) subsets in the tumor microenvironment. TAMs display a wide range of phenotypes. SPP1⁺ TAMs perform multiple protumor functions, including promoting metastasis, angiogenesis, and immunosuppression. This subset facilitates tumor-cell migration via SPP1–CD44 interaction, and mediates ECM degradation through upregulation of MMP9 and MMP12. In addition, SPP1⁺ TAMs secrete VEGF and PDGF to drive neovascularization. CCL18⁺ TAMs are believed to represent a terminally differentiated state within the SPP1⁺ macrophage lineage and further enhance immunosuppression by recruiting naïve T cells and promoting their differentiation into Tregs. C1Q⁺ TAMs may be associated with favorable responses to immune-checkpoint blockade. FCN1⁺ TAMs are considered intermediates in the monocyte-to-macrophage differentiation trajectory, with the potential to give rise to both C1Q⁺ and SPP1⁺ TAM subsets.TREM2⁺ macrophages exhibit a strong positive correlation with the accumulation of regulatory T cells and a negative correlation with cytotoxic CD8⁺ T cell infiltration. Functionally, this subset suppresses CD8⁺ T cell activity through lipid uptake and Treg interaction. Angiogenesis-associated TAMs (Angio-TAMs) are marked by high expression of HIF1A and SLC2A1, which drives tumor angiogenesis. Another vascular-related TAM subset is the LYVE1⁺ TAMs, which form multicellular perivascular clusters near blood vessels. IL-6 signaling promotes CCR5 expression in these TAMs, facilitating their localization. Additionally, elevated expression of HO-1 in LYVE1⁺ PvTAMs contributes to immune exclusion of CD8⁺ T cells from the tumor microenvironment. Although IFN-responsive TAMs (IFN-TAMs) exhibit a phenotype resembling M1-like macrophages, they demonstrate strong immunosuppressive functions by suppressing T cell activity through tryptophan-degrading metabolic pathways and recruiting regulatory T cells to promote immune evasion.

Rare, functionally specialized TAM states also link macrophage plasticity to neural remodeling. In Lewis lung carcinoma models, scRNA-seq identifies a TUBB3⁺ TAM subset within the macrophage lineage that co-expresses neurogenesis-associated genes such as *SHANK, MAP1B*, and *SYT13*. Higher TUBB3⁺ TAM abundance correlates with increased nociceptive behaviors in tumor-bearing mice, and Smad3 emerges as a transcriptional regulator initiating macrophage-to-neuron transition (MNT) programs [139]. These data provide one of the first functional connections between TAM plasticity, tumor-associated neurogenesis, and cancer pain. Representative examples and functional assays for these metabolically, checkpoint-, and neurogenesis-associated TAM subsets are summarized in Table 2 and Fig. 2.

#### Metabolic reprogramming of CAFs

Integrated analyses combining scRNA-seq with metabolomics have revealed that CAFs profoundly influence tumor progression through diverse metabolic programs that vary across tumor types.

In PDAC, loss of the histone H3K36 trimethyltransferase SETD2 in tumor cells reprograms stromal fibroblasts into a lipid-rich CAF state: SETD2 deficiency induces ectopic H3K27ac deposition and activates BMP2 signaling, driving differentiation of ABCA8a⁺ lipid-laden CAFs that transfer lipids to cancer cells and fuel mitochondrial oxidative phosphorylation and accelerated tumor growth [140]. In CRC, multi-omics single-cell and spatial profiling have identified distinct metabolic CAF subsets, including an SFRP2⁺ CAF cluster enriched for ECM-remodeling genes, positioned adjacent to Treg infiltrates and associated with poor prognosis; although functional perturbation is limited, this spatial co-localization supports a role in matrix stiffening and immunosuppression [141]. In soft-tissue sarcoma (STS), a glycolysis-dependent CAF subset (glyCAFs) was identified via scRNA-seq, shown experimentally to secrete CXCL16 through a GLUT1 pathway and establish physical T cell exclusion barriers; glycolysis inhibition reduces glyCAF accumulation at tumor margins, enhances T cell entry, and improves chemotherapy delivery [142]. Together, these examples illustrate how metabolically specialized CAF states can shape immune exclusion and treatment response, highlighting stromal metabolism as a potential therapeutic target.

## Molecular and multi-omic determinants of CAF and TAM functional plasticity

### Metabolic reprogramming

#### Metabolic reprogramming of TAMs

TAMs dynamically shape the immunosuppressive TME through diverse metabolic programs.

##### Carbohydrate metabolism

Transcriptomic and metabolic-flux analyses show that pro-inflammatory MHC-II⁺ TAMs exhibit impaired tricarboxylic-acid (TCA) cycle activity due to reduced expression of the mitochondrial complex II subunit SDHB, whereas reparative MHC-II⁻ TAMs display enhanced glycolysis and oxidative phosphorylation [143]. Lactate acts as a central metabolic intermediate with dual effects: in MHC-II⁻ TAMs, it activates arginase-1 (ARG1) and engages the kynurenine pathway to suppress CD8⁺ T cell activity, while in MHC-II⁺ TAMs, lactate inhibits AMPK signaling and dampens metabolic activity [143]. These findings highlight how a shared metabolite can differentially tune TAM phenotypes and immune suppression.

##### Lipid metabolism

Lipid handling is another key axis of TAM-mediated immune regulation. In glioblastoma, single-cell and multi-omics profiling identifies lipid-laden macrophages (LLMs) enriched in mesenchymal GBM; phagocytosis of cholesterol-rich myelin debris induces an LLM phenotype that shuttles myelin-derived lipids to tumor cells through an LXR/ABCA1-dependent mechanism, supporting the heightened metabolic demands of MES-GBM [144]. In PDAC, integrated scRNA-seq, spatial transcriptomics, and metabolomics reveal M1-like macrophages tightly linked to lipid metabolism and inversely correlated with tumor-cell abundance; within this compartment, IRF7 and RPS18 emerge as prognostic genes, and IRF7-mediated repression of RPS18 limits exosomal transfer of ribosomal proteins to PDAC cells, thereby constraining ILF3 expression, lipid metabolism, proliferation, and invasion [145]. Additional work in PDAC indicates that KRAS-driven GM-CSF can stimulate TAM citrate catabolism via PI3K/Akt, culminating in immunosuppressive ornithine accumulation, further connecting oncogenic signaling to macrophage-centered metabolic reprogramming [111, 131].

##### Amino-acid and nucleotide metabolism

Amino-acid pathways add a further layer of complexity. In GC, scRNA-seq with functional validation shows that MSR1^hi^ macrophages undergo arginine- and proline-pathway reprogramming driven by AMPK/mTOR activation, adopt M2-like phenotypes, and promote tumor progression [146]. In glioma, microglia-derived TAMs are enriched for “cytoplasmic translation” signatures, whereas monocyte-derived TAMs preferentially express “purine nucleotide metabolism” pathways [147]. Purine metabolism marks protumor, terminally differentiated TAMs, correlates with poor prognosis and diminished response to immune-checkpoint blockade [148], and similar purine-rich, low–antigen-presentation TAM clusters have been identified in liver metastases alongside glycolytic, OXPHOS, lipid, and amino acid–metabolic clusters [148]. Hypoxia-driven, proangiogenic TAM subsets with convergent metabolic signatures have also been reported in PDAC and ovarian cancer [111, 131]. Collectively, these data outline a TAM-centered network linking energy fueling, immune suppression, and therapy resistance, and provide a rationale for combinatorial strategies that pair metabolic modulators (e.g., IDO1-pathway inhibitors, LXR-targeting agents) with immunotherapy.

### Epigenetics: crosstalk between chromatin architecture and immune regulation

Epigenetic dysregulation is increasingly recognized as a key driver of tumor progression and immune escape in the TME, acting through chromatin remodeling, transcriptional reprogramming, and multilayered interaction networks [149]. Although many pathways are broadly conserved across cancer types, their outcomes remain highly tissue- and context-specific.

Three-dimensional chromatin architecture provides a compelling example of functionally validated epigenetic remodeling. In BC, scATAC-seq combined with DNA FISH revealed decompaction of pericentromeric human satellite II (hSATII) loci in stromal and tumor cells, forming a novel interaction domain (DRISR) enriched for SASP-associated transcription factor motifs [150]. Functional assays demonstrated that DRISR activation induces pro-inflammatory cytokines, indicating that spatial chromatin reorganization can directly promote SASP signaling and tumor progression [150]. In LUAD, epigenetic–metabolic coupling functionally shapes CAF behavior: CAFs with elevated nicotinamide *N*-methyltransferase (NNMT) undergo metabolic reprogramming that increases H3K4me3 levels and upregulates ECM-related genes, including collagens and integrins, thereby enhancing metastatic capacity in vitro and in vivo [151]. These studies provide direct evidence that chromatin remodeling in CAFs can drive protumor remodeling of the TME.

More broadly, single-cell ATAC-seq in colorectal tissue reveals progressive gains in chromatin accessibility at T cell-exhaustion–associated loci along the transition from healthy colon to cancer, changes that inversely correlate with local DNA methylation [152]. While these patterns underscore the importance of methylation–accessibility imbalance in immune remodeling, the functional impact on T cell suppression remains incompletely defined. Intercellular epigenetic crosstalk is also evident in intrahepatic cholangiocarcinoma (ICC): vascular CAFs (vCAFs) secrete IL-6, which activates EZH2-dependent histone methylation in tumor cells and promotes malignancy, while tumor-derived exosomal miR-9-5p feeds back to limit IL-6 expression in vCAFs, forming a reciprocal—but still partially resolved—regulatory loop [42].

DNA methylation reprogramming substantially influences TAM function. Classically, DNA methylation is best known for gene silencing via promoter hypermethylation [153], but in a 4T1 TNBC model, tumor cells impose widespread methylation changes on TAMs: cytokines such as TGF-β, IFN-γ, and CSF1 activate transcription factors including FOSL2, STAT1, and RUNX3, and hypomethylation of the Cd274 promoter induces PD-L1 expression in TAMs, providing direct mechanistic evidence that methylation-driven changes can enforce immune suppression [154]. In glioma, IL4I1 expression in TAMs inversely correlates with promoter methylation and increases with tumor grade, although the demethylation mechanism remains unclear [155]; pan-cancer single-cell analyses further show that IL4I1⁺ macrophages promote immunosuppression via tryptophan degradation and facilitate Treg recruitment, with mechanistic strength varying across tumor types [94].

At the histone-modification level, tumor-promoting histone lactylation in TAMs has been demonstrated in PTEN/p53-deficient genetically engineered mouse models of PCa. PI3K inhibitor treatment reduces lactate production in cancer cells, lowers H3K18lac levels in TAMs, and enhances their phagocytic activity; this effect is further amplified when androgen-deprivation therapy is combined with anti-PD-1 immunotherapy [156]. In metastatic castration-resistant PCa, histone lactylation in TAMs promotes tumor growth, and scRNA-seq analyses of biopsies link glycolytic activity to suppressed TAM phagocytosis [156]. Together, these findings position epigenetic remodeling—including 3D chromatin architecture, DNA methylation, and histone lactylation—as a critical layer coupling stromal and myeloid states to immune evasion, and highlight opportunities to integrate epigenetic modulators with CAF- and TAM-targeted therapies.

### CITE-seq: a bimodal approach integrating transcriptomes and surface proteins

CITE-seq enables simultaneous measurement of transcriptomes and surface proteins by tagging cell-surface markers with oligonucleotide-labeled antibodies, allowing highly multiplexed multimodal profiling at single-cell resolution [157]. In glioblastoma and GL261 models, combining CITE-seq with scRNA-seq confirmed TMEM119—originally identified at the RNA level—as a microglia-specific marker in RTM-TAMs, validating its protein-level specificity [158]. CITE-seq also revealed important discordances between mRNA and protein abundance: in human breast cancer, PD-L1 and PD-L2 transcripts appeared enriched mainly in Inflam-TAMs by scRNA-seq, whereas CITE-seq showed that PD-L1/PD-L2 proteins were distributed across Inflam-TAMs, LA-TAMs, and Reg-TAMs, indicating post-transcriptional regulation and cautioning against inferring checkpoint expression from RNA alone [129]. In NSCLC, CITE-seq-based integration identified an immune-activation module (LCAM^hi^) composed of PDCD1⁺CXCL13⁺ exhausted T cells, IgG⁺ plasma cells, and SPP1⁺ macrophages, which correlated with high tumor mutational burden, TP53 mutations, and improved response to immune-checkpoint blockade [98]. Together, these studies illustrate how CITE-seq refines immune-cell classification and exposes functional protein-level regulation that is invisible to transcriptome-only approaches.

### INs-seq: functional sequencing of intracellular proteins and transcriptomes

INs-seq (intracellular-sequencing) is a recently developed single-cell method that enables large-scale, parallel profiling of intracellular protein activity together with RNA transcriptomes, allowing metabolically active immune-cell subsets to be defined based on their intracellular functional states [159]. Using INs-seq in tumor models, investigators systematically mapped arginase-1–expressing cells and discovered a previously unrecognized Arg1⁺Trem2⁺ metabolic-regulatory (Mreg) macrophage population, delineating its defining markers, metabolic activity, and regulatory pathways [159]. This approach illustrates how functional proteomic readouts can refine myeloid classification beyond traditional surface markers and transcript-based definitions.

The rise of single-nucleus RNA-seq (snRNA-seq) has further enabled high-resolution characterization of human tumors, including CAF subsets and their transcriptional programs [160–162]. In BC, CAFs were classified into four major programs—ADH-F, IMM, MYO, and NRT—based on gene-expression profiles [160]. The MYO program, marked by high ACTA2, overlaps with myCAF-like states and is enriched for mesodermal-development and Wnt-signaling genes, whereas ADH-F, IMM, and NRT programs broadly align with previously described iCAF-like phenotypes; notably, post-therapy samples showed expansion of ADH-F and IMM programs, indicating therapy-induced stromal remodeling and iCAF program enrichment [36, 160].

### Multiple omics technologies

Multi-omics analyses of immune and stromal cells in the TME are increasingly revealing their functional heterogeneity and dynamic regulatory networks. In PDAC, integration of scRNA-seq, metabolomics, and multicolor immunohistochemistry delineated four major TAM subsets, including a proliferative tissue-resident macrophage (rMφ) population that elevates intracellular deoxycytidine (dC) and suppresses deoxycytidine kinase (dCK), thereby limiting gemcitabine uptake by tumor cells and promoting fibrosis and immunosuppression via TGF-β and collagen secretion [163]. Targeted depletion of rMφs in transgenic mouse models reduces tumor burden, identifying these cells as potential chemosensitization targets [163]. Subsequent PDAC studies combining scRNA-seq, spatial transcriptomics, and exosomal metabolomics have uncovered immune–metabolic networks in which M1-like macrophages restrict ribosomal-protein transfer via the IRF7/RPS18 axis and curb lipid metabolism and metastatic behavior in tumor cells [145], while GM-CSF–PI3K–AKT signaling and lactate metabolism polarize metabolically “educated” ARG1⁺ACLY⁺TXNIP⁺ TAMs (TEMs), providing an intervention window to reprogram macrophage metabolic plasticity [164].

In *Clonorchis sinensis*–associated intrahepatic cholangiocarcinoma (iCCA), integrated scRNA-seq, whole-exome sequencing, metabolomics, and spatial transcriptomics revealed marked enrichment of fatty-acid biosynthesis and fatty-acid synthase (FASN) pathways [165]. Spatial data showed closer co-localization of malignant cells with TAM-like macrophages in *C. sinensis*–associated iCCA than in non-Clonorchis tumors, and elevated FASN and free fatty acids promoted immunosuppression and tumor progression; pharmacologic FASN inhibition reversed the immunosuppressive TME and potentiated anti-PD-1 therapy [165]. These studies illustrate how multi-omics pipelines can link specific metabolic circuits in CAFs and TAMs to therapeutic vulnerabilities.

Multilayered omics integration has also been applied to CAF lineages. Foster et al. combined epigenomic, transcriptomic, and proteomic datasets to define three conserved CAF meta-programs—steady-state–like (SSL), mechano-responsive (MR), and immunomodulatory (IM)—across tissues, with MR-CAFs differentiating into IM and SSL subsets through YAP/TAZ-mediated mechanotransduction [166]. Immune-checkpoint blockade perturbed this balance, suggesting that targeting CAF plasticity may represent a therapeutic strategy [166]. In esophageal squamous cell carcinoma(ESCC), combined scATAC-seq, scRNA-seq, and ChIP-seq mapped progressive chromatin changes along fibroblast trajectories from normal mucosal fibroblasts to NAFs, iCAFs, and myCAFs, with stepwise activation of transcription factors such as TCF21, BATF, and NFYB and increased accessibility at collagen and contractility genes [167]. Parallel analyses of TAMs identified regulators, including FOS, ZEB1, and CEBPA and accessible regions near angiogenesis-related (VEGFA) and immune-checkpoint (PD-L1) loci. While these data provide robust evidence of chromatin remodeling and lineage progression, direct functional assays of the individual fibroblast and macrophage subsets and their full spatiotemporal dynamics remain to be established [167].

Harmonization of large-scale spatial multi-omics datasets has begun to resolve CAF–TAM organization at the tissue scale. In iCCA, investigators integrated imaging mass cytometry, spatial proteomics, spatial transcriptomics, multiplex immunofluorescence, scRNA-seq, bulk RNA-seq, and proteomics to profile over 1.06 million cells [168]. Spatial topology—including cell deposition, community architecture, and intercellular communication—strongly correlated with patient outcome, and five spatial iCCA subtypes with distinct immunostromal signatures were defined; CD163^hi^ M2-like tissue-resident macrophages directly suppressed CD8⁺ T cell activity and associated with poorer survival, and a deep-learning model trained on spatial TME features accurately predicted prognosis from a single 1 mm² section [168]. Using imaging mass cytometry, Hong et al. generated a complementary spatial single-cell proteomic atlas of iCCA and independently identified five reproducible spatial TME subtypes with distinct stromal and immune compositions, including TLS-like–high, CD57⁺ epithelial–high, granulocyte–high, CAF-high/CD163^hi^ macrophage–high, and Pan-CK⁺ epithelial–high configurations [169]. This spatially resolved framework clarifies how different CAF- and macrophage-enriched niches structure the iCCA microenvironment and provides a blueprint for precision therapeutic stratification.

## Intercellular crosstalk: orchestrating CAF–TAM interactions in the tumor microenvironment

**Fig. 3:**
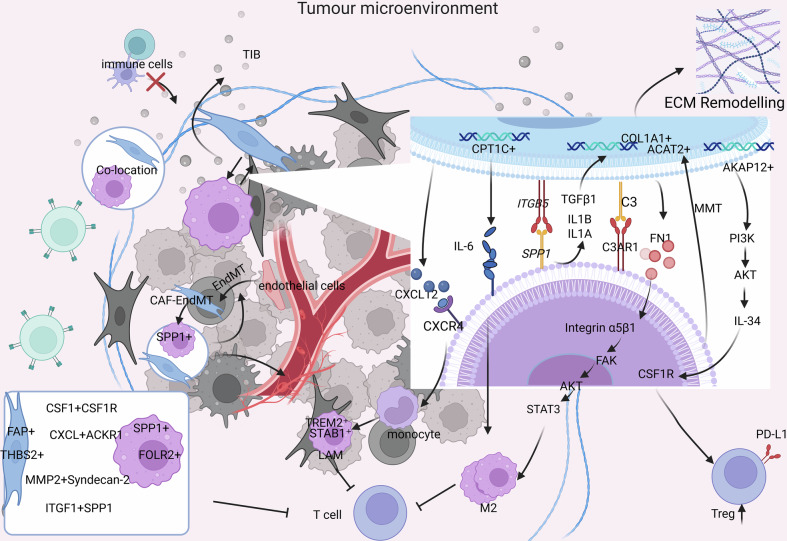
Multidimensional mechanisms of CAF–TAM crosstalk in shaping the immunosuppressive tumor microenvironment (TME). In the TME, cancer-associated fibroblasts (CAFs) and tumor-associated macrophages (TAMs) interact through diverse molecular pathways to orchestrate the formation of an immunosuppressive microenvironment. TAMs are the predominant immune cell population in regions enriched with CAFs, and these two cell types are spatially co-localized with extensive mutual interactions. Together, they stimulate extracellular matrix (ECM) remodeling and promote the formation of tumor immune barriers (TIBs) at the tumor margins, thereby limiting immune infiltration into the tumor core. CAFs can arise from endothelial cells via endothelial-to-mesenchymal transition (EndMT), and this CAF-EndMT subset further enhances EndMT progression and angiogenesis through interactions with SPP1⁺ TAMs. At the molecular level, CAFs induce the differentiation of monocytes into STAB1⁺TREM2⁺ lipid-associated macrophages (LAMs) via the CXCL12–CXCR4 signaling axis, thereby promoting immune evasion by suppressing CD8⁺ T cell clonal expansion. CAF-derived CPT1C⁺ immunosuppressive cytokines, such as IL-6, drive macrophage polarization toward an M2-like phenotype. In addition, fibroblast-expressed ligand C3 specifically activates various C3 receptors (e.g., C3AR1) on macrophages, facilitating direct intercellular communication. Fibroblasts also express ITGB5, the receptor for SPP1 secreted by TAMs, enabling reciprocal interaction. AKAP12⁺ CAFs activate macrophage CSF1R signaling through the PI3K/AKT/IL-34 axis, thereby promoting the expansion of PD-L1⁺ regulatory T cells. Moreover, CAF-secreted fibronectin (FN1) triggers the integrin α5β1-FAK-AKT-STAT3 cascade in macrophages, reinforcing M2 polarization through a dual mechanism. Conversely, TAMs act on CAFs through multiple pathways. For instance, TAM-derived SPP1 binds to CAF-expressed ITGF1, remodeling the tumor microenvironment. M2-polarized TAMs can directly transdifferentiate into tumor-promoting ACTA2⁺COL1A1⁺ CAFs via MMT, a process driven by Smad3 signaling. Additionally, SPP1⁺ TAMs secrete a triad of cytokines-IL1A, IL1B, and TGFB1-that activate ECM-related gene expression (e.g., COL1A1 and COL3A1) in FAP⁺ CAFs, leading to the development of a fibrotic, connective tissue-rich microenvironment. CAF–TAM communication is also mediated through multiple ligand–receptor pairs, including CSF1–CSF1R, CXCL–ACKR1, MMP2-Syndecan-2, and ITGF1-SPP1.

Inside the TME, CAFs and TAMs cooperate to construct a profoundly immunosuppressive niche via multiple, interlocking molecular circuits. TAMs constitute the dominant immune population adjacent to CAF-rich zones, highlighting intense physical and functional reciprocity [170]. CAFs and TAMs are spatially co-localized, enabling extensive bidirectional communication through direct membrane-bound interactions as well as paracrine and exosomal signaling. Pan-cancer integration further shows that fibroblasts interact with macrophages far more frequently than with T or B cells [84], and emerging data even position macrophages themselves as an abundant alternative source of CAF-like cells within tumors [171]. Major CAF–TAM ligand–receptor axes and their functional consequences are summarized in Table 3 and Fig. 3.

**Table 3:** Key signaling pathways and functional effects of CAF–TAM interactions.

Signaling Axis	Sender	Receptor	Molecular mechanism	Cancer type	Assay	Spatial location
CXCL12–CXCR4	FAP^+^ iCAF	Monocyte	Monocyte differentiation into STAB1^+^ TREM2^+^ LAM, Immunosuppression, T cell suppression	Triple-negative breast cancer [13]	scRNA-seq	Not reported
	CFD^+^ iCAF	TAM	Immunomodulation	Pan-cancer [84]	scRNA-seq+ST	Not reported
C3-C3aR1/ITGB2/ITGAM/ITGAX	FD^+^ iCAF	TAM	Not reported	Pan-cancer [84]	scRNA-seq+ST	Not reported
C3-C3aR	CD34^+^ iCAF	Monocyte	Monocyte recruitment, immunosuppressive phenotype, and reduced T cell infiltration	Melanoma [177]	scRNA-seq	Not reported
C3-C3aR1	THBS2^+^ mCAFs	SPP1^+^ TAMs	Leading to immunotherapy resistance	Gastric cancer [202]	scRNA-seq	Co-localized
SPP1-ITGB5	SPP1^+^ TAM	MMP11^+^myCAF	Not reported	Pan-cancer [82]	scRNA-seq+ST	Not reported
SPP1-ITGF1	APOC1^+^/SPP1^+^ TAM	CAF	Reshaping the tumor microenvironment	Hepatocellular carcinoma [185]	scRNA-seq	Not reported
IL-6/IL-6R	*CPT1C*^*+*^*CAFs*	M2-TAM	Promote M2 polarization	Gastric cancer [179]	scRNA-seq	Not reported
PI3K/AKT/IL-34-CSF1R	*AKAP12*^*+*^ *CAF*	TAM	Promote M2 polarization, immunosuppression	Triple-negative breast cancer [182]	scRNA-seq+CyTOF+ST	Co-localized
TIMP1–CD63	CAF	TAM	Reshaping the tumor microenvironment	Hepatocellular carcinoma [185]	scRNA-seq	Not reported
SPP1–CD44	CAF (EndMT)	SPP1^+^ TAM	Facilitate intratumoral angiogenesis	Pan-cancer [83]	scRNA-seq	Not reported
SDC2-MMP2	SPP1^+^ TAM	CAF	Tumor proliferation and metastasis	Colorectal cancer [95]	scRNA-seq	Not reported
TGFβ1-Smad3	M2-TAM	ACTA2^+^COL1A1^+^ CAF	Macrophage-myofibroblast transition (MMT)	Non-small cell lung cancer [186]	scRNA-seq+ChIP-seq	Not reported
CSF1–CSF1R, CXCL–ACKR1	FAP^+^ CAF	SPP1^+^ TAM	Not reported	Prostate cancer [188]	scRNA-seq+ST	Co-localized
MDK-NUPR1, FGF - SPP1	POSTN^+^ CAF	SPP1^+^ TAM	ATP metabolic, glycolytic, and NAD metabolic processes, promoting fibroblast proliferation	Head and neck squamous cell carcinoma [189]	scRNA-seq	Not reported
MMP1-FN1, MMP13-IL1RN, MMP9-ACTA2	SPP1^+^ TAM	POSTN^+^ CAF	ECM organization, cell-substrate adhesion and focal adhesion	Head and neck squamous cell carcinoma [200]	scRNA-seq	Not reported
FN1-Integrin α5β1	FAP^+^ CAF	TAM	FAK-AKT-STAT3 drives M2 polarization, immunosuppression	Breast cancer [183]	scRNA-seq	Not reported

*CAF* cancer-associated fibroblast, *iCAF* inflammatory cancer-associated fibroblast, *myCAF* myofibroblastic cancer-associated fibroblast, *TAM* tumor-associated macrophage, *LAM* lipid-associated macrophage, *M2* alternatively activated macrophages, *ECM* extracellular matrix, *EndMT* endothelial-to-mesenchymal transition, *scRNA-seq* single-cell RNA sequencing, *ST* spatial transcriptomics, *ChIP-seq* chromatin immunoprecipitation sequencing, *CyTOF* cytometry by time-of-flight.

CAF–TAM dialog is mediated by both direct contact and a rich cocktail of soluble mediators. CAFs release interleukins (e.g., IL-1β, IL-6, IL-8, IL-10, and IL-33), chemokines (CXCL1–10, CXCL12/SDF-1, CXCL16, CCL2, CCL3, CCL5, CCL7, CCL20, and CCL26), and factors such as TGF-β, PGE2, IDO, LIF, VEGF, TNF, nitric oxide, and Chi3L1, which act synergistically to recruit macrophages into the stroma and polarize them toward tumor-supportive phenotypes [6, 172–176].

### CAF-driven TAM recruitment and M2-like polarization

Across cancers, CAFs steer TAM differentiation and function through a set of recurrent ligand–receptor circuits. In TNBC and oral squamous cell carcinoma (OSCC), scRNA-seq shows that FAP⁺ iCAFs induce monocytes to adopt a STAB1⁺TREM2⁺ lipid-associated macrophage (LAM) identity via the CXCL12–CXCR4 axis; these LAMs are enriched in anti-PD-1–refractory tumors and curtail CD8⁺ T cell clonal expansion, thereby intensifying immune escape [13]. A pan-tissue meta-analysis of 249,000 fibroblasts from 73 datasets corroborates that CXCL12 is most abundant in CFD⁺ inflammatory fibroblasts, which preferentially pair with macrophages through CXCR4 and also activate the complement ligand C3 to engage macrophage receptors C3AR1 and ITGB2/ITGAM/ITGAX [84]. Together, these data highlight CXCL12–CXCR4 and C3-based circuits as conserved CAF-driven modules that recruit and imprint macrophages toward immunoregulatory states.

Complement-dependent recruitment is further exemplified by CD34⁺ iCAFs in MEL and HNSCC, which secrete C3 to attract monocytes via C3-C3aR and skew them toward immunosuppressive phenotypes, thereby shaping T cell infiltration [177]. Because CD34⁺ CAFs uniquely express high C3, this subset may serve as a tractable biomarker or stromal target, and C3a–C3aR blockade in tumor-bearing mice reduces tumor growth and F4/80⁺ macrophage density [177]. In GC, extracellular matrix–associated POSTN⁺ CAFs (eCAFs) co-localize with M2 macrophages, and CPT1C⁺ CAFs help establish a dense, IL-6–rich immunosuppressive stroma that reinforces M2-like polarization [178, 179].

Recent work has identified two CAF-derived secreted proteins—IGFBP7 and PRSS23—as key regulators of macrophage infiltration and polarization in GC. IGFBP7, highly expressed in CAFs and inducible by TGF-β, enhances cancer progression by activating the FGF2–FGFR1–PI3K–AKT axis; CAF-derived IGFBP7 increases FGF2 transcription and secretion, and FGFR1-high macrophages respond by adopting an M2-like, immunosuppressive phenotype, with increased stromal infiltration and poor prognosis [180]. Complementing this, PRSS23, a serine protease enriched in CAFs, elevates FGF2 expression and secretion, and FGF2 is required for PRSS23-mediated macrophage recruitment and TAM polarization, as shown by rescue experiments in PRSS23-depleted models. PRSS23–FGF2 signaling strongly correlates with canonical M2/TAM markers (CD163, MRC1, MSR1) and reduced overall survival [181]. Together, these findings establish IGFBP7 → FGF2 and PRSS23 → FGF2 as convergent CAF-driven pathways that remodel the immune landscape by enhancing TAM infiltration and skewing macrophage polarization toward pro-tumoral states.

Beyond these flagship examples, multi-omics studies have uncovered additional CAF-derived circuits that promote TAM recruitment and M2-like programming—including AKAP12⁺ CAF–IL-34–CSF1R–PI3K–AKT signaling in TNBC, FAP⁺ CAF–RARRES2/CMKLR1 and fibronectin–integrin α5β1–FAK–AKT–STAT3 pathways, and ST3GAL4-dependent sialylated glycans on CAFs engaging Siglec-7/9/10/15 on monocytes to drive immunosuppressive TAM differentiation [14, 182–184]. These axes further underscore the dominant role of the CAF secretome and stromal glycosylation in orchestrating TAM recruitment and polarization (Table 3).

### TAM-mediated reverse regulation of CAF activation and function

While CAFs shape TAM phenotypes, TAMs can reciprocally regulate CAF activation and function. In HCC, APOC1⁺SPP1⁺ TAMs secrete SPP1, which engages ITGB1 on CAFs, while CAF-derived TIMP1 binds CD63 on tumor cells and TAMs [185]. This SPP1–ITGB1 / TIMP1–CD63 intercellular circuit remodels the TME, reinforcing fibroblast activation and promoting HCC progression. In parallel, FAP⁺ CAFs can interact with naïve T cells via CXCL12–CXCR4, a stromal–immune axis that likely contributes to immune-checkpoint inhibitor resistance in this context [185] (Table 3).

Beyond paracrine signaling, TAMs themselves can serve as a source of CAFs through macrophage-myofibroblast transition (MMT). In NSCLC, M2-polarized TAMs were shown to convert into pro-tumorigenic ACTA2⁺COL1A1⁺ CAFs under Smad3 control [186]. Single-cell trajectory analysis revealed a temporal progression from bone marrow-derived macrophages to fibroblast-like cells with stepwise upregulation of α-SMA and type I collagen [186]. ChIP-seq demonstrated direct Smad3 binding at ACTA2 and COL1A1 promoters, and metabolic profiling identified increased LDHA- and GLS-driven glycolysis and glutaminolysis as metabolic support for this transition. Pharmacologic Smad3 inhibition effectively blocked MMT and reduced tumor growth and metastasis [186]. Together, these findings delineate a temporally ordered reprogramming route through which M2-like TAMs generate ACTA2⁺COL1A1⁺ CAFs, establishing a critical reverse regulatory axis within the CAF–TAM interaction network.

### CAF–TAM cooperation in ECM remodeling and immunosuppression

Growing evidence indicates that CAFs and TAMs coordinately remodel the ECM and enforce immunosuppression through a limited set of recurrent stromal circuits. Single-cell multi-omics in CRC and other solid tumors has pinpointed FAP⁺/POSTN⁺ CAFs and SPP1⁺ TAMs as a central axis: FAP⁺ fibroblasts engage SPP1⁺ macrophages via CSF1–CSF1R and CXCL–ACKR1 signaling, while SPP1⁺ TAMs secrete IL1A, IL1B, and TGFB1 to induce COL1A1/COL3A1 and other ECM-synthesis genes in FAP⁺ CAFs, driving desmoplastic matrix deposition and a stiff, immunosuppressive microenvironment [14, 96, 187, 188]. In LUAD and CRC liver metastases, similar CAF–TAM ligand–receptor networks (including FGF1, CSF1, PGF, TGFB3, and TIMP1 from CAFs and SPP1, FN1, APOE in macrophages) reprogram monocyte-derived macrophages toward inflammatory–fibrotic, lipid-dysregulated states and are associated with Treg enrichment and CD8⁺ T cell exclusion [103, 109]. Together, these findings position FAP⁺/POSTN⁺ CAF–SPP1⁺ TAM interactions as a core module coupling ECM remodeling to immune suppression.

Longitudinal single-cell atlases further reveal that reciprocal crosstalk between POSTN⁺ CAFs and SPP1⁺ TAMs intensifies with tumor progression. In advanced LUAD, POSTN⁺ fibroblasts increasingly regulate the metabolic properties of SPP1⁺ macrophages, while SPP1⁺ macrophages secrete MMP1 and MMP9 to enhance ECM organization and cell–matrix adhesion in POSTN⁺ fibroblasts, jointly promoting fibrotic niche formation and malignant reprogramming [189, 190]. Additional macrophage subsets, such as FOLR2⁺ tissue-resident macrophages and APOC1⁺APOE⁺ metastasis-associated macrophages that express COL6A1/COL6A2 and signal to myofibroblasts via ITGA11, have been implicated in fibrosis, stromal reorganization, and collagen deposition in kidney, prostate, and breast cancer, but most evidence remains correlative [152, 191, 192] (Table 3).

Spatial multi-omics has begun to resolve how these circuits organize ECM-rich, immune-excluded niches. In HCC, paired scRNA-seq and spatial transcriptomics identified a “tumor immune barrier” (TIB) at the invasive margin, where hypoxia-induced SPP1⁺ macrophages interact with CAFs and malignant hepatocytes to drive ECM remodeling and restrict immune-cell infiltration into the tumor core; disruption of this SPP1⁺ TAM–CAF–tumor triad, combined with anti-PD-1 therapy, enhances treatment efficacy [193]. In early LUAD and ER⁺ breast cancer, THBS2⁺ or myofibroblastic CAFs co-localize with M2-like or CCL2⁺ TAMs and are associated with T cell exclusion and increased expression of exhaustion markers [194, 195], while spinal ependymoma data implicate PGF–CCL2⁺ TAM interactions in shaping pro-apoptotic and fibrotic phenotypes [133]. These spatially resolved studies underscore that CAF–TAM ECM circuits are not uniformly distributed but instead assemble into discrete, prognosis-relevant stromal niches.

Exosomes provide an additional layer of ECM-focused communication between fibroblasts and macrophages. In silica-induced pulmonary fibrosis, pyroptotic macrophages release exosomes enriched in profibrotic miRNAs that drive fibroblast-to-myofibroblast transition via TGF-β signaling, increasing α-SMA expression, ECM production, and collagen deposition [196]. Conversely, in skin wound healing, fibroblast-derived exosomes tune the temporal dynamics of macrophage polarization and accelerate the transition from inflammatory to reparative states [197], whereas macrophage-derived exosomes carrying miR-142-3p can suppress epithelial and fibroblast activation by targeting TGFβR1 and attenuate downstream profibrotic programs [198]. Complementing these findings, snRNA-seq studies in fibrotic tissues identified CXCL4-instructed SPP1⁺Fn1⁺Arg1⁺ macrophages as central hubs that engage fibroblasts through SPP1–integrin, fibronectin-mediated, and Sema3-family signaling, driving myofibroblast activation and fibrosis progression [199, 200]. Although many of these insights derive from non-malignant fibrotic models, they delineate conserved exosome- and cytokine-based CAF–TAM circuits that are readily co-opted by tumors to sustain desmoplasia and immunosuppression (Table 3).

### CAF–TAM crosstalk in tumor angiogenesis

CAF–TAM interactions contribute to angiogenesis by coupling endothelial remodeling with macrophage reprogramming. A pan-cancer survey across 10 tumor types identified an endothelial-derived CAF subset (CAF-EndMT) generated via endothelial-to-mesenchymal transition (EndMT); these cells co-express CD44, CD31, and ACTA2 and are regulated by RGS5, PLVAP, and VWF [83]. CAF-EndMTs preferentially engage SPP1⁺ TAMs, accelerating ongoing EndMT and neovascular growth and associating with poor clinical outcome [83]. In BC, tumor-secreted TGF-β upregulates CXCR4 on CCR2⁺ monocytes, steering them toward CXCL12-rich perivascular CAFs; the resulting CXCR4⁺ TAMs assemble a pro-metastatic vascular niche that facilitates tumor-cell intravasation and systemic dissemination [200].

In spinal ependymomas, integrated scRNA-seq and ATAC-seq analyses predicted ligands that shape the transcriptional program of CD44⁺ TAMs, including angiogenesis-associated CTGF, SFRP2, and ANGPT1 expressed by fibroblasts, endothelial cells, and pericytes [133]. Within anaplastic ependymoma, CD44⁺ TAMs showed enriched interactions with these stromal and vascular compartments through ligand–receptor pairs such as WNT5A–ROR2, JAG1–NOTCH3, and ANGPT2–TEK, all functionally linked to vascular remodeling [133]. Collectively, these circuits illustrate how CAFs, TAMs, and endothelial/perivascular cells cooperate to promote angiogenesis, with additional CAF–TAM–endothelial axes summarized in Table 3.

### CAF–TAM metabolic interactions

Beyond structural cues, CAF–TAM coupling is fine-tuned by bidirectional metabolic rewiring that sustains an immunosuppressive milieu. In HNSCC, POSTN⁺ CAFs release FGF ligands that signal to SPP1⁺ TAMs, activating programs in ATP production, glycolysis, and NAD synthesis and thereby fueling CAF proliferation and tightening stromal–immune metabolic symbiosis [190]. These data highlight a metabolically coupled POSTN⁺ CAF–SPP1⁺ TAM axis that reinforces both ECM remodeling and immune suppression.

In GC, LOX⁺ fibroblasts within hypoxic regions induce monocyte differentiation into M2-like macrophages via IL-6/IL-6R signaling, establishing a self-amplifying circuit in which hypoxia drives LOX⁺ fibroblasts, IL-6 production, and M2 macrophage accumulation, collectively promoting tumor progression and an immunosuppressive niche [201]. Single-cell transcriptomic profiling of primary and metastatic ESCC revealed a metabolically active stromal–immune axis centered on POSTN⁺ myofibroblasts and APOC1⁺APOE⁺ macrophages, both enriched within metastatic niches [61]. POSTN⁺ CAFs exhibited strong ECM-remodeling and lipid-processing signatures, whereas APOC1⁺APOE⁺ macrophages showed enhanced lipid uptake and transport, supporting models in which a tumor-cell–dominated metabolic program sustains primary growth, while a macrophage-driven lipid–adhesion module promotes matrix remodeling and lymph-node metastasis [61]. Together, these findings underscore CAF–TAM metabolic crosstalk as a key driver of pro-metastatic microenvironments in ESCC and other cancers, with additional glycolytic, amino-acid, and lipid-handling circuits detailed in Table 3.

### CAF–TAM spatial co-localization and tumor niches

Within the TME, CAFs and TAMs occupy shared microniches that form multilayered regulatory webs. Spatially resolved analyses in CRC show that macrophages and CAFs are tightly co-localized within metastatic CRC (mCRC) lesions [103]. scRNA-seq and spatial transcriptomics demonstrate that FAP⁺ CAFs nestle beside SPP1⁺ TAMs at the invasive front, creating a malignant circuit that stiffens the matrix and impedes T cell ingress [14]. This pathologic architecture persists in CRC liver metastases, underscoring its role as a conserved pro-metastatic hub [103].

BC exhibits subtype-specific CAF–TAM topologies. In the tumor core, CD163⁺/CD206⁺ M2-like TAMs cluster with αSMA⁺ myCAFs to form a canonical T cell–excluding niche, whereas at luminal-A margins, spatial transcriptomics reveals Detox-iCAFs neighboring FOLR2⁺ protective macrophages [59]. Tumor-derived cues can convert Detox-iCAFs into ECM-myCAFs, which in turn skew TAMs toward a TREM2⁺ phenotype and reinforce T cell exclusion [59]. Spatial pairing of AKAP12⁺ CAFs with CSF1R⁺ TAMs further amplifies Smad3-driven immunosuppressive signaling in TNBC [182].

Metastatic contexts reveal analogous CAF–TAM circuitry. In gastric-cancer peritoneal metastasis, THBS2⁺ myCAFs attract tissue-resident macrophages through platelet-response protein 2 and, via the C3-C3AR1 axis, convert F13A1⁺ TRMs into SPP1⁺ TAMs; blockade of C3AR1 restores CD8⁺ T cell entry and enhances ICB efficacy [202]. In GBM, an atypical CAF-like population collaborates with mesenchymal glioma stem cells (GSCs) and M2-like TAMs to build a three-way supportive niche: GSC-derived PDGF and TGF-β activate CAFs, which release EDA⁺ fibronectin that signals through TAM TLR4, inducing IL-10/ARG1 upregulation and M2 polarization; these TAMs then secrete OPN and HGF to sustain GSC stemness via PI3K–Akt–MET, locking the niche into a self-reinforcing loop [203].

Pan-tumor spatial meta-analyses confirm a conserved repertoire of CAF–TAM layouts. Pairing of SPP1⁺APOE⁺ TAMs with CTHRC1⁺GREM1⁺ myCAFs drives suppressive ECM deposition and EMT and correlates with poor survival [204]. Similar LOX⁺ CAF–M2-TAM coupling in GC hastens matrix stiffening [192], whereas in MEL, C3aR⁺ TAMs abut CD34⁺ CAFs and enforce CXCL12–CXCR4-mediated immune exclusion [177]. TNBC spatial maps likewise reveal durable co-localization of AKAP12⁺ CAFs with CSF1R⁺ macrophages [182]. Collectively, these patterns highlight CAF–TAM spatial co-organization as a key determinant of stromal architecture and immune exclusion (Table 3 and Fig. 3).

### Antitumor effects of CAF–TAM interactions

CAF–TAM crosstalk is bidirectional: besides their well-known protumor roles, CAFs can, under specific cues, elicit antitumor programs by re-educating TAMs. Slit2 secreted by CAFs binds Robo1 on macrophages, driving them toward an M1-like state and enhancing tumor-cell phagocytosis [205]. In parallel, Slit2 increases MMP expression in M1-polarized TAMs, accelerating collagen breakdown and softening the fibrotic stroma; in BC models, this axis reduces matrix rigidity and limits tumor growth, while clinical data link high Slit2 levels to improved survival and lower CD163⁺ TAM infiltration [205].

## Spatiotemporal dynamics of CAF and TAM heterogeneity

Spatiotemporal heterogeneity within the TME has become a central focus of cancer biology. Among stromal components, CAFs and TAMs are two of the most abundant and plastic populations, and single-cell and spatial multi-omics increasingly show that their phenotypes are profoundly shaped by tumor stage, microanatomical localization, and reciprocal crosstalk.

### Temporal dynamics of CAF and TAM states

Longitudinal profiling indicates that CAF and TAM programs evolve in waves during tumor progression. In PDAC, integrated high-dimensional proteomics and transcriptomics placed apCAFs adjacent to precancerous PanIN lesions; as PanIN advanced, CAF signaling shifted from inflammatory to proliferative modes, and mouse models revealed stage-specific CAF “waves”, in which early subsets were gradually supplanted by populations enriched for growth-factor, inflammatory and myofibroblast markers such as ACTA2 and Tagln [206, 207]. In parallel, a distinct periductal senescent CAF (SenCAF) population accumulates over time in hypoxic ductal niches [208, 209]. The senescence-associated secretory phenotype (SASP) of pancreatic fibroblasts induces immunosuppressive markers in macrophages and impairs T cell effector function, providing a temporal link between fibroblast senescence, TAM reprogramming and immune suppression [208].

In BC, spatially resolved transcriptomics identified ten FAP⁺ CAF clusters that could be grouped into eco-cellular types (ECTs) with distinct temporal behavior. FAP⁺ myCAFs (ECM-myCAF, TGFβ-myCAF, IFNαβ-myCAF) are positioned near tumor cells, whereas Detox-iCAFs localize around vasculature and are relatively enriched in ductal carcinoma in situ (DCIS) but reduced in invasive lesions. Cancer cells promote the differentiation of Detox-iCAFs into Wound-myCAFs and ECM-myCAFs via DPP4–YAP1–dependent mechanisms, and Detox-iCAFs, IL-iCAFs and IFNγ-iCAFs recruit monocytes and induce FOLR2⁺ TAM phenotypes, while TGFβ-myCAFs at invasive fronts foster TREM2⁺ TAMs and NKG2A⁺ regulatory NK cells [59]. Similarly, another BC study clustered CAFs into two archetypal subtypes based on mutually exclusive PDPN (pCAF) and S100A4 (sCAF) expression; during tumor progression, sCAFs underwent dynamic remodeling, with MHC-II⁺ antigen-presenting subsets becoming dominant by week 4, and the S100A4/PDPN ratio serving as a prognostic classifier in independent cohorts [41]. In TNBC mouse models, apCAFs emerged mainly in late-stage disease and systemic spread, supporting a model in which antigen-presenting fibroblast programs are temporally induced during advanced progression [41].

TAMs also undergo temporal reprogramming. In ccRCC, macrophage phenotypes differ markedly between early, locally advanced, and metastatic stages: early tumors harbor pro-inflammatory, chemokine-rich macrophages, whereas late-stage lesions show increased anti-inflammatory and immunosuppressive signatures, indicating stage-dependent functional shifts [114]. In NSCLC, scRNA-seq of tumor-associated leukocytes demonstrated that tissue-resident macrophages (TRMs) aggregate near tumor cells during early progression, promoting EMT, invasiveness, and Treg activation, but decline as tumors advance, while monocyte-derived macrophages (MDMs) expand and dominate late-stage lesions. Spatial tracing revealed that tumor cells initially cluster around TRMs but later migrate outward as MDM-rich regions expand, reflecting dynamic remodeling of TAM composition over time [210]. Similar origin-dependent patterns are seen in glioma, where microglia-derived macrophages localize to the tumor periphery, while bone marrow-derived Mo-TAM_inf (IBA1⁺/CXCL3⁺) accumulate around necrotic and perivascular zones and contribute to angiogenesis [211]. In MEL, monocyte-derived HO-1⁺ TAMs preferentially localize at invasive margins via NRF2 activation coordinated by NF-κB1–CSF1R–C3aR signaling, promoting an immunosuppressive and proangiogenic microenvironment [132]. In immunocompetent GBM models, TAM redistribution mirrors vascular remodeling and hypoxia: MDMs initially cluster around organized vessels but, as vasculature becomes tortuous and hypoperfused, migrate toward vessel-sparse hypoxic regions, illustrating tight coupling between macrophage positioning and evolving microenvironmental stress [212].

### Spatial organization of CAF programs and stromal niches

CAF states exhibit pronounced spatial patterning that underpins distinct stromal niches. Pan-cancer integrative analyses identify CTHRC1 as a defining marker of ECM-associated CAFs enriched at tumor–normal interfaces; spatiotemporal mapping shows that eFibro_CTHRC1 cells increase from non-tumorous to tumor regions and co-localize with SLPI⁺ profibrotic macrophages, forming spatially organized profibrotic ecotypes confirmed by multiplex IHC [213].

In PDAC and BC, CAF spatial architecture tracks with functional specialization. In PDAC, SenCAFs accumulate in hypoxic periductal regions [208, 209], while apCAFs reside near early PanIN lesions, and myofibroblastic CAFs dominate more advanced desmoplastic areas [206, 207, 214]. In BC, FAP⁺ myCAFs cluster around tumor nests, whereas Detox-iCAFs align with perivascular regions as described above [59], and spatial proteomic analysis of ER⁺ disease demonstrates that myCAFs and M2-like macrophages are positioned close to tumor cells in T cell–excluding rims, while iCAFs expressing CXCL12 shape perivascular zones that recruit Tregs and M2-like TAMs and impede cytotoxic T cell infiltration via the CXCL12–CXCR4 axis [195].

Tumor type–specific CAF lineages also segregate spatially along the metastatic cascade. In colorectal neuroendocrine-tumor liver metastasis (CRNENLM), liver-metastatic CAFs (LM-CAFs) show heightened ECM-remodeling signatures—for example, COLEC11_mCAFs that support hepatic colonization—whereas primary-lesion CAFs (PL-CAFs) display stronger inflammatory and angiogenic programs, reflecting differential stromal organization between primary and metastatic sites [215]. In OSCC, RGS4⁺ mCAF1 (myofibroblast-derived) dominates primary tumors, whereas COMP⁺ mCAF2 (fibroblastic-reticular-cell-derived) is enriched in metastatic lymph nodes and correlates with extranodal extension [216]. In HCC, spatial proteogenomic analyses identified two matrix prototypes—CAF-FAP and CAF-C7—enriched in intratumoral stroma and fibrotic rims, respectively. CAF-FAP co-localizes with PDCD1⁺ exhausted T cells in intratumoral inflammatory centers and is associated with T cell suppression, whereas CAF-C7 co-localizes with SPP1⁺ macrophages in peritumoral wound-healing zones, forming PDGFRA–PDGFC-mediated fibrotic loops that restrict immune infiltration [217].

Single-cell and spatial imaging in LUAD identified four major CAF clusters, two of which (MYH11⁺αSMA⁺ and FAP⁺αSMA⁺) are linked to T cell exclusion [189]. The MYH11⁺αSMA⁺ population forms a thin layer encapsulating tumor aggregates in early-stage disease and produces type IV collagen, whereas FAP⁺αSMA⁺ CAFs assemble thicker stromal patches around tumor nests at advanced stages and deposit type XI/XII collagens, creating distinct ECM barriers [189]. In HNSCC, IL-11⁺ iCAFs spatially associate with inflammatory monocytes under IL-1β/TNF-α–driven NF-κB signaling, whereas CCL19⁺ fibroblastic-reticular cell (FRC)-like fibroblasts are enriched in immune “hotspot” HPV⁺ tumors and engage tertiary lymphoid structures via noncanonical NF-κB signaling [218]. Collectively, these observations underscore that CAF programs are not uniformly distributed but organize into discrete spatial prototypes linked to immune exclusion, angiogenesis, and metastatic colonization.

### Spatial and metabolic plasticity of TAMs

TAM function is strongly conditioned by spatial context and local metabolic cues. Classical IHC studies in CRC showed that CD68⁺ macrophage infiltration along tumor fibers correlates with a favorable prognosis, whereas intraepithelial CD68⁺ macrophages associate with poor outcome [219, 220]. Subsequent analyses using scRNA-seq and IHC clarified that macrophages occupy distinct anatomical niches—tumor center (TC) versus invasive front (TF)—with the CD163⁺/CD68⁺ ratio significantly higher at TF, correlating with lymphovascular invasion, advanced TNM stage, and EMT-associated circulating tumor cells [221]. Single-cell data further revealed that pro-inflammatory Inflam-TAMs cluster in luminal regions rich in necrosis and neutrophils, whereas immunosuppressive IFN-TAMs localize near CXCL13⁺ T cells and CXCL10/11-expressing tumor foci, suggesting that interferon signaling drives immune escape in specific microanatomical zones [214].

Metabolic programs reinforce this spatial compartmentalization. In primary CRC, LA-TAMs exhibit high oxidative phosphorylation signatures, whereas in liver metastases, the same LA-TAM cluster re-engages tryptophan, arginine, proline, alanine, and glutamine metabolism, demonstrating site-specific metabolic rewiring that supports metastatic outgrowth and chemoresistance [122]. Single-cell and spatial studies in CRC liver metastasis further showed that CAF-derived ligands (e.g., FGF1, CSF1, PGF, TGFB3, and TIMP1) reshape TAM phenotypes toward inflammatory fibrosis and dysregulated lipid handling, characterized by SPP1, FN1, and APOE expression, accompanied by Treg enrichment and CD8⁺ T cell depletion in the metastatic niche [103].

Across additional tumor types, TAM origin and localization jointly shape function. In NSCLC, the temporal TRM-to-MDM shift described above is mirrored by spatial redistribution from tumor–stromal interfaces to deeper, hypoxic tumor regions [210]. In glioma and GBM, microglia-derived TAMs localize to tumor borders, whereas bone marrow-derived TAMs accumulate in necrotic and perivascular zones and later track emerging hypoxia and vascular disorganization, contributing to angiogenesis and immunosuppression [211, 212]. In MEL and other solid tumors, monocyte-derived HO-1⁺ TAMs or other stress-adapted subsets preferentially reside at invasive margins, where they integrate hypoxia, complement, and CSF1R signals to promote vascular remodeling and immune evasion [132]. Lee and colleagues used an elegant in vivo photoconversion lineage-tracing approach combined with scRNA-seq, spatial mapping, flow cytometry and functional assays to resolve temporally and spatially distinct TAM populations and their interactions with stromal fibroblasts. They show that circulating monocytes continuously replenish a highly dynamic monocyte-derived TAM (mdTAM) compartment that is enriched in the tumor core, whereas a resident-like TAM (rTAM) population is stably localized at the tumor–normal interface and spatially co-localizes with fibroblasts. Importantly, newly infiltrating mdTAMs diverge along two fate trajectories distinguished by MHC-II expression: MHC-II⁺ mdTAMs exhibit enhanced endocytosis, FcγR-mediated phagocytosis and antigen-presentation capacity, while MHC-II⁻ mdTAMs are transcriptionally distinct and less phagocytic. These functional differences were demonstrated experimentally by ex vivo antigen-presentation assays (Eα peptide), immune-complex uptake (Ova-IC), cytokine readouts and flow-cytometric phenotyping [222].

Mechanistically and spatially relevant to CAF–TAM crosstalk, rTAMs at the tumor boundary show upregulated PDGF and TGFβ ligands and form a ring-like architecture with PDGFRβ⁺ fibroblasts, consistent with a role in fibroblast activation and capsule/ECM maintenance. Anti-PD-L1 treatment preferentially affects newly infiltrating mdTAMs (rather than rTAMs), skews mdTAM differentiation toward the MHC-II⁺ fate, restores inflammatory gene programs, increases mdTAM–CD4⁺ T cell CXCL9/10 signaling, and thereby generates an IFNγ-CXCL9/10 feed-forward loop— demonstrating subset-specific and temporally dependent responses to immune-checkpoint blockade. These findings provide direct functional and spatiotemporal evidence that TAM identity, function and drug responsiveness are coupled to both time-of-entry and tissue location [222].

Collectively, these findings indicate that CAFs and TAMs exhibit remarkable spatiotemporal plasticity and co-evolution, generating distinct ecological niches that govern immune infiltration, angiogenesis, and metastatic dissemination. Decoding these dynamics through integrated spatial multi-omics will be essential for reconstructing the CAF–TAM interactome in four dimensions (space and time) and for identifying therapeutic opportunities to selectively remodel stromal and myeloid compartments.

### Targeted therapy of TAMs

#### Depleting and redirecting TAMs: from bulk removal to controlled recruitment

Because TAMs are predominantly tumor-supportive in most TMEs, early therapeutic efforts focused on depleting these cells, whereas more recent strategies aim to limit their recruitment and selectively reshape their functions. These approaches largely converge on three axes: CSF1–CSF1R signaling, chemokine-guided trafficking, and phagocytic or surface checkpoints.

One major avenue is blocking the CSF1–CSF1R axis, which is central to monocyte-to-macrophage differentiation and TAM survival. Small-molecule or antibody-mediated CSF1R inhibition reduces TAM infiltration and promotes a shift toward less immunosuppressive myeloid states. Pexidartinib, a CSF1R/KIT/FLT3 inhibitor first approved in tenosynovial giant cell tumor, has been extended to trials in prostate, lung, breast, and hematologic cancers, exemplifying this depletion-oriented strategy [223]. Other agents, such as the multi-kinase inhibitor sulfatinib (HMPL-012), which also targets VEGFR1–3 and FGFR1, and the CSF1-neutralizing antibody lacnotuzumab (MCS110), similarly aim to disrupt CSF1–CSF1R signaling and thereby limit TAM accumulation [223, 224]. Overall, these agents illustrate that sustained suppression of CSF1R can decompress TAM-rich niches, although compensatory myeloid influx and systemic toxicities remain key challenges.

A second strategy interrupts chemokine-guided trafficking of monocytes and macrophages into tumors. Dual CCR2/CCR5 antagonists such as BMS-813160 are being evaluated in NSCLC and HCC, with the goal of reducing monocyte ingress into the TME and preventing their differentiation into M2-like TAMs [225]. In parallel, disruption of the CXCL12–CXCR4 axis using NOX-A12, a PEGylated RNA aptamer targeting CXCL12, has advanced from phase I to phase II trials, often in combination with other agents [225]. Combination regimens—for example, bevacizumab plus the CCL2 inhibitor mNOX-E36—illustrate a broader concept in which chemokine blockade is layered onto anti-angiogenic therapy to simultaneously limit macrophage influx and tumor vascularization [225]. Collectively, these approaches seek not only to reduce TAM numbers but also to blunt the continuous recruitment of immunosuppressive myeloid cells into tumor sites.

Beyond recruitment, a third line of attack focuses on directly targeting TAM surface molecules and phagocytic checkpoints. Among the best-characterized antigens are TREM2 and SPP1 [128, 226]. In mouse models, anti-TREM2 antibodies—alone or combined with PD-1 blockade—reduced tumor burden and reprogrammed TAMs toward a more pro-inflammatory state [227]. SPP1-directed RNA aptamers similarly curtailed the recruitment of CD206⁺/F4/80⁺ macrophages and slowed disease progression [226]. Interestingly, clinical observations are not uniformly negative: in HNSCC, high TREM2⁺ TAM infiltration has been associated with improved outcome [228], underscoring context dependence and the need for careful patient stratification.

In parallel, reactivating macrophage phagocytosis via the CD47–SIRPα checkpoint has emerged as a complementary strategy. Tumor-expressed CD47 delivers a “don’t-eat-me” signal that restrains macrophage-mediated clearance [229]. The first-in-class anti-CD47 antibody magrolimab (Hu5F9-G4) has shown early activity in phase I studies across ovarian, colorectal, prostate, and hematologic malignancies [230], with phase II trials ongoing in breast, colorectal, and head and neck cancers. Parallel approaches include SIRPα-blocking fusion proteins such as evorpacept (ALX-148), currently tested in HER2-positive GC, and bispecific antibodies like IBI-322 (anti-CD47/PD-L1), which aim to couple phagocytic activation with checkpoint inhibition [160, 231]. Conceptually, these agents do not simply deplete TAMs but convert them into active effector cells capable of engulfing tumor cells and presenting antigen. Together, CSF1R blockade, chemokine interception, and phagocytic/surface-checkpoint targeting provide complementary levers to reduce TAM burden, limit the influx of new immunosuppressive macrophages, and reawaken the phagocytic and inflammatory potential of those that remain.

#### TAM reprogramming—restoring pro-inflammatory immunity

Therapeutic strategies increasingly emphasize the reprogramming of TAMs rather than their depletion, aiming to shift macrophages from immunosuppressive M2-like states toward pro-inflammatory, antigen-presenting phenotypes. Recent work highlights that metabolic and signaling cues can profoundly reshape TAM identity. For instance, inhibition of APOC1 promotes ferroptosis-associated conversion of M2 macrophages into M1-like cells, thereby remodeling the immunosuppressive TIME and enhancing anti-PD-1 efficacy in HCC [232].

Engaging costimulatory pathways provides another robust route to TAM re-education. CD40 agonists convert suppressive TAMs into cytotoxic, antigen-presenting cells capable of restoring antitumor immunity. The prototypical agent APX005M (sotigalimab) demonstrated encouraging responses when combined with chemotherapy or nivolumab in PDAC [233], and additional agonists such as SEA-CD40 and CDX-1140 are advancing clinically in NSCLC, MEL, and ovarian cancer [234, 235].

Innate-immune activation through TLR engagement likewise repolarizes macrophages toward an inflammatory state [236]. Nanoparticle delivery of a dual TLR7/8 agonist (vR848) drove M1-like differentiation and inhibited tumor growth in CRC models [237]. Clinically relevant TLR agonists—including poly I:C (TLR3), MPLA (TLR4), imiquimod (TLR7) and CpG oligodeoxynucleotides (TLR9) [238]—have demonstrated TAM activation in BC [239] and HNSCC [240]. Together, these lines of evidence outline a coherent framework in which metabolic cues, CD40 signaling and TLR engagement act as complementary levers to reinstate antitumor macrophage functions.

#### Metabolic reprogramming of TAMs—disrupting lipid and amino-acid dependencies

A second major therapeutic logic centers on exploiting the metabolic plasticity of TAMs. Multi-omics studies show that perturbing glucose–lipid circuitry can redirect macrophage fate. In BRCA-deficient TNBC models, PARP inhibition remodels glucose and lipid metabolism under SREBF1 control, altering TAM differentiation; co-administration of PARP blockade with CSF1R inhibitors produced synergistic antitumor immunity in vivo [241]. Similarly, inhibition of fatty-acid oxidation with etomoxir significantly suppressed tumor growth by interrupting TAM metabolic support [233, 242].

Surface-directed modulation of metabolic regulators represents another axis of intervention. The humanized anti-CD44 antibody RG7356, which influences monocyte differentiation through ERK1/2, has shown acceptable safety in early clinical testing [230]. Attempts to disrupt amino-acid catabolism—most notably tryptophan degradation via IDO1 inhibition—have yielded mechanistic insights into TAM-centered immunometabolic regulation, despite the negative outcome of the ECHO-301 trial [243].

Beyond small molecules, engineered cellular metabolism is emerging as a transformative direction. HER2-targeted CAR-macrophages eradicated tumors in HER2⁺ ovarian cancer models [244, 245], and early-phase trials are now evaluating their activity with or without anti-PD-1 therapy [246]. A nanoparticle-hydrogel scaffold has further enabled localized delivery of CD133-directed CAR-Ms, achieving site-restricted macrophage reprogramming while minimizing systemic toxicity [247]. Collectively, these studies illustrate how metabolic rewiring—via inhibitors or engineered macrophages—can reinvigorate antitumor immunity by breaking TAM lipid and amino-acid symbiosis.

### Targeting CAFs

#### Targeted therapies for FAP

Fibroblast activation protein (FAP)—a signature surface marker of CAFs—has become a central entry point for TME-oriented interventions. Optimized SynCon FAP DNA vaccines overcome host immune tolerance in multiple murine models (TC-1, Brpkp110, TSA), inducing robust FAP-specific CD8⁺/CD4⁺ T cell responses and markedly suppressing tumor growth; co-administration with tumor-antigen DNA vaccines further augments antitumor efficacy, although these combinations remain at the preclinical stage [248]. Clinically, FAP-directed chimeric antigen receptor T cells (CAR-T-FAP) are being evaluated in metastatic pleural mesothelioma, and dual-targeting constructs such as Nectin4-7.19/FAP-12 CAR-T cells have eradicated pulmonary metastases in NSG models, underscoring the versatility of FAP-targeted cellular immunotherapy [249–253].

Beyond direct killing, FAP is also emerging as a diagnostic and theranostic hub. The quinoline-derived tracer Ga-FAPI-04 offers superior PET/CT contrast compared with conventional ¹⁸F-FDG, allowing more accurate discrimination between malignant and normal tissues and enabling image-guided stromal targeting [254–257]. Complementing these approaches, near-infrared photo-immunotherapy (NIR-PIT) directed against FAP selectively ablates FAP⁺ cells within dense ECM; EC models demonstrate rapid, FAP-specific cytotoxicity in vitro and in vivo with minimal collateral damage, highlighting NIR-PIT as a promising modality for CAF depletion [258, 259].

#### CAF pharmacological reprogramming

Pharmacological reprogramming of CAFs is particularly advanced in PDAC, where CAFs are tightly linked to pancreatic stellate cells (PSCs). Quiescent PSCs store retinoic acid (RA), whereas oncogenic and inflammatory cues deplete RA and drive their conversion into pro-tumorigenic, myofibroblast-like CAFs [260]. All-trans retinoic acid (ATRA) restores PSC quiescence and remodels the stroma toward a less supportive state: in preclinical PDAC models, ATRA plus gemcitabine significantly restrains tumor growth [261], and a Phase Ib study confirmed that ATRA with gemcitabine/nab-paclitaxel is safe and well tolerated in patients with advanced unresectable PDAC [262].

Beyond RA signaling, vitamin D has emerged as a complementary stromal reprogramming agent. By modulating Wnt/β-catenin and related pathways, vitamin D attenuates the pro-tumorigenic activity of CAFs and increases PDAC sensitivity to chemotherapy [263]. Together, these data support a therapeutic paradigm that converts CAFs from a pathogenic to a more quiescent, therapy-permissive state rather than simply depleting them.

#### ECM remodeling inhibition strategies

Targeting ECM remodeling represents a second major avenue for stromal intervention. PEGPH20—a PEGylated recombinant human hyaluronidase—selectively degrades hyaluronic acid (HA), a key ECM component. In PDAC mouse models, HA depletion lowers interstitial fluid pressure, improves vascular perfusion, and enhances intratumoral drug distribution [264, 265]. The angiotensin-receptor blocker losartan similarly mitigates TGF-β signaling, dampens CAF activation, and reduces deposition of HA and collagen, thereby facilitating chemotherapy delivery and sensitizing tumors to immunotherapy [266].

Focal adhesion kinase (FAK) has emerged as a central mechanotransduction node coupling CAF contractility, matrix stiffness, and immune exclusion [267]. In PDAC, the FAK inhibitor VS-4718 alleviates stromal fibrosis and immunosuppression, restoring immune surveillance and increasing sensitivity to immune-checkpoint blockade and radiotherapy [267, 268]. Combination regimens with anti-PD-L1 drive extensive ECM remodeling, promote deep CD8⁺ T cell infiltration, and markedly augment the efficacy of PD-L1 blockade [269, 270]. Additional strategies target CAF-derived MMPs; for example, hybrid liposomes functionalised with MMP2–responsive peptides preferentially accumulate in PDAC tissue and suppress ECM synthesis by pancreatic stellate cells, thereby improving gemcitabine penetration and antitumor activity [271]. Collectively, these approaches establish a multi-pronged framework for breaching the stromal barrier in PDAC.

#### Targeting CAF-related signaling pathways

A third strategy focuses on CAF-centered signaling pathways, with the TGF-β axis as a pivotal hub. TGF-β drives the emergence of LRRC15⁺ CAFs that promote immune evasion and is closely linked to poor responses of PDAC patients to PD-L1 blockade [262]. Dual inhibition of TGF-β and PD-1/PD-L1 reprograms LRRC15⁺ CAFs and reverses stroma-remodeling gene signatures in breast cancer models [272]. Clinically, fresolimumab—a neutralizing antibody against TGF-β1/2/3—has shown tumor-suppressive activity with an acceptable safety profile [273, 274], while the TGF-β receptor I inhibitor galunisertib (LY2157299) exerts antitumor effects as monotherapy and in combination with gemcitabine in PDAC and HCC [275, 276]. The bifunctional fusion protein bintrafusp alfa concurrently targets TGF-β and PD-L1, binds TGF-β1 with high affinity, and markedly suppresses tumor growth in preclinical models [277].

In parallel, disrupting the CXCL12–CXCR4 axis addresses CAF-driven immune exclusion. CXCL12 secreted by CAFs recruits monocytes and skews them toward immunosuppressive phenotypes via CXCR4, maintaining a tumor-permissive milieu [13]. In metastatic breast cancer models, CXCR4 antagonism with AMD3100 enhances CTL infiltration, limits stromal expansion, and dismantles suppressive niches [278]. This concept is now being tested in metastatic PDAC (AMD3100 plus anti-PD-1) [279] and in trials evaluating the CXCR4 inhibitor BL-8040 (motixafortide) combined with pembrolizumab and liposomal irinotecan to reprogram the tumor immune microenvironment via multi-pathway modulation [280].

#### Targeting CAF metabolism

Targeting CAF metabolism offers a complementary route to disrupt the energetic co-dependence between tumor cells and stroma. In CRC models, knockdown of tumor-cell-intrinsic hexokinase (HK) and glucose-6-phosphatase (G6Pase)—both regulated by CAF-derived cues—markedly suppresses tumor growth. Building on these findings, concomitant inhibition of HK and G6Pase with agents such as metformin and polydatin has shown encouraging antitumor effects [281].

Emerging drug-development platforms allow more precise metabolic intervention. Bispecific antibodies that simultaneously engage multiple metabolic checkpoints exert stronger inhibitory effects, whereas proteolysis-targeting chimeras (PROTACs) enable selective degradation of individual metabolic enzymes, adding a new layer of control over stromal–tumor metabolism [281]. In parallel, the tyrosine-kinase inhibitor NT157, which blocks both IGF-1R and STAT3 signaling, modulates tumor cells and their surrounding stroma. In CRC models, NT157 not only restrains tumor progression directly but also reduces CAF-mediated secretion of protumor cytokines, attenuating the metabolism–immunity axis that fuels tumor growth [282].

#### Targeting CAF–TAM interactions

A final therapeutic avenue seeks to directly dismantle CAF–TAM crosstalk, guided by multi-omics dissection of their interaction networks. In relapsed/refractory diffuse large B-cell lymphoma (RR-DLBCL), integrative scRNA-seq, ECM proteomics, and 3D co-culture models revealed a dual mode of action for FGFR1 inhibitors (FGFR1i): FGFR1i reprogram FGFR1⁺ CAFs into an inflammatory phenotype that upregulates CCL2 and CXCL12, thereby recruiting antitumor TAMs and skewing them toward a phagocytic state, while simultaneously promoting deposition of immunostimulatory proteoglycans such as decorin and lumican to remodel the ECM niche [283].

The hypoxia-inducible factor (HIF) axis constitutes a second regulatory hub. Conditional deletion of HIF-2α in CAFs markedly reduces M2-like TAM and Treg infiltration, and pharmacologic blockade with the HIF-2α antagonist PT2399—originally developed for renal-cell carcinoma—increases tumor susceptibility to immunotherapy [284]. Further work on MMT identifies Smad3 as a master driver of CAF emergence from TAMs: in Lewis LUAD models, the Smad3 inhibitor SIS3 attenuates MMT and significantly slows tumor progression [186]. Additional targets include G-protein-coupled receptor 30 (GPR30), an estrogen receptor highly expressed in CAFs that mediates TAM influx and M2 polarization; genetic ablation of GPR30 reduces invasion and metastasis in PCa [243].

In gastric-cancer peritoneal metastasis, THBS2⁺ myofibroblastic CAFs attract tissue-resident macrophages via thrombospondin-2 and engage the complement C3-C3AR1 axis to convert them into SPP1⁺ TAMs; pharmacologic C3AR1 inhibition breaks this circuit and enhances the efficacy of combined immune-checkpoint blockade and chemotherapy [202]. In TNBC, intratumoral AKAP12⁺ CAFs orchestrate spatial M2 polarization of macrophages through IL-34–CSF1R signaling, and interruption of this pathway markedly improves responsiveness to PD-1 blockade [182]. Collectively, these studies highlight CAF–TAM interaction nodes—FGFR1, HIF-2α, Smad3-driven MMT, complement C3–C3AR1, and IL-34–CSF1R—as actionable levers for multicellular re-engineering of the TME.

## Advancing single-cell multi-omics: bridging challenges to clinical translation

### Technical and translational constraints

Despite the transformative capacity of single-cell multi-omics to decode the TME, several fundamental constraints still impede broad clinical adoption. High cost and stringent specimen requirements—including the need for fresh tissue, adequate cell viability, and specialized processing—restrict most studies to relatively small discovery cohorts, while validation often reverts to more economical bulk RNA-seq [285]. Although frozen material can be used for snRNA-seq, it generally provides lower-resolution information than fresh single-cell workflows, further limiting large-scale, uniform analyses [286].

A second barrier lies in integrating multimodal datasets. Newer protocols can capture epigenomic, proteomic, and metabolic layers alongside transcriptomes, but the differing signal-to-noise properties and potential cross-modality conflicts demand robust, scalable computational frameworks capable of extracting coherent biological meaning [23]. A third limitation is technical ceiling: insufficient sequencing depth and coverage can erase low-abundance transcripts and rare states, compromising cell-type annotation and downstream functional inference [287]. Moreover, transcriptomic read-outs alone frequently under-represent metabolic plasticity or short-lived immune-activation states that are central to CAF and TAM function.

Collectively, these hurdles underscore the need for cost-efficient platform design, improved sample-preservation and processing workflows, and next-generation algorithms for cross-omics fusion. Addressing these bottlenecks will be essential if single-cell multi-omics is to realize its full potential in clarifying CAF–TAM dynamics and guiding precision oncology [285–287].

## Conclusion

TAMs and CAFs jointly shape the tumor microenvironment by reinforcing immunosuppressive niches, remodeling tissue architecture, and modulating treatment response. Single-cell and spatial studies have revealed substantial heterogeneity within both lineages, but they also show that many proposed CAF and TAM subtypes lack validated functions, leading to a proliferation of labels and datasets that do not always translate into mechanistic insight.

Three pivotal obstacles now define the next phase of the field. First, static omics snapshots cannot resolve the spatiotemporal nuances of CAF–TAM metabolic exchange. Second, current therapeutic modalities—such as FAP-CAR-T cells and CSF1R blockade—are limited by off-target toxicities and compensatory resistance mechanisms. Third, the delivery efficiency of engineered tools, including ECM-degrading nanoparticles, remains suboptimal in the mechanically stiff TME.

Overcoming these hurdles will require cross-disciplinary programs—typified by a “Dynamic TME Atlas” approach—that combine million-cell single-cell multi-omics, real-time imaging and longitudinal clinical cohorts to train graph neural networks capable of predicting patient-specific target combinations. Emerging tools such as PLATO, an AI- and microfluidics-driven spatial proteomics platform that profiles thousands of proteins at 25-µm whole-tissue resolution [288], and MiP-seq, a subcellular-resolution in situ sequencing method that simultaneously maps DNA, RNA, proteins and metabolites at near single-base precision [289], exemplify this trajectory.

Advanced technologies will remain important, but their primary role should be to clarify core biological principles, not to generate ever more complex subtype catalogs. Integrating existing datasets into coherent mechanistic models will help the field converge on a smaller number of functionally meaningful CAF and TAM states. By shifting from descriptive heterogeneity toward experimentally validated mechanisms, it should become possible to design more precise strategies to modulate the stromal–immune axis and ultimately improve patient outcomes.

## Supplementary information

Reproducibility_Checklist

## Data Availability

The data that support the findings of this study are available from the corresponding author upon reasonable request.
